# Artificial Neptune balls: Superadhesive biomimetic networks for broad-size microplastics capture and removal

**DOI:** 10.1126/sciadv.aeg0819

**Published:** 2026-08-05

**Authors:** Haeleen Hong, Byeunggon Kim, Mesbah Ahmad, Orlin D. Velev

**Affiliations:** Department of Chemical and Biomolecular Engineering, North Carolina State University, Raleigh, NC 27695-7905, USA.

## Abstract

The cleanup of persistent microplastics (MPs) from aquifers requires the capture and removal of a broad range of MP sizes and shapes. Conventional methods such as filtration and centrifugation are inefficient in removing such a broad range of particle sizes. We designed a class of biomimetic cleaners inspired by natural systems—including “*Sargassum* rafts” that trap MPs within their branched thalli and “Neptune balls” formed from seagrass. The cleaners are in the form of porous meshes and balls made of biopolymers such as alginate and chitosan. The biopolymers are reprocessed morphologically into soft dendritic colloids (SDCs). The SDCs are consolidated in a honeycomb-like internal network surrounded by a hierarchically fibrillar outer layer. This mesh architecture enables adsorption of nano- and microscale particles via van der Waals and electrostatic interactions while physically trapping millimeter-scale particles into the net openings. Similar cleaners from architected sustainable materials could serve as scalable systems for efficient removal of diverse MPs from aquatic environments.

## Teaser

Cleanup of broadly sized microplastic particles is achieved by replicating principles found in natural seagrass formations.

## INTRODUCTION

The global production and extensive use of synthetic polymers have resulted in the accumulation of degraded plastic waste in oceans and freshwater systems, causing dire environmental consequences (*1*–*3*). Many widely used synthetic plastics are highly resistant to both abiotic and biotic degradation, making them extremely persistent in the environment. Over time, environmental factors such as ultraviolet radiation, oxidation, salinity, and mechanical forces like wave shear break large plastic pieces into smaller fragments (*4*), classified as microplastics (MPs; 0.1 μm to 5 mm in diameter) or, when degraded further, as nanoplastics (NPs; <0.1 μm) (*5*–*7*). These plastic particles can adsorb pollutants or serve as substrates for bacterial biofilms (*1*, *8*). Owing to their high surface-to-volume ratio, smaller particles tend to adsorb greater amounts of pollutants and microbes (*9*, *10*). Furthermore, NPs and MPs can enter the food chain and pose elevated risks to ecosystems and human health (*9*, *11*). NPs and MPs are now ubiquitous in aquatic environments, including oceans, rivers, lakes, and wastewater effluents (*12*).

The removal of the plastic particles from aquatic systems remains a major global challenge. The larger fragments from polymer products can be captured by filtration. Such elutriation methods are typically effective for MP particles of diameter >50 μm (*13*, *14*). However, the removal of the microscale plastic particles from large volumes of water or open environments by conventional filtration is impractical and cost-inefficient (*15*, *16*). Density-based flotation and velocity-based separation can remove particles of sizes from a few micrometers to tens of micrometers (*17*). The filtration methods are even less suitable for the removal of MP particles of micrometer size or smaller size range, which leads to pore clogging and membrane fouling, causing excessive pressure drops and reduced permeance (*18*, *19*). The removal of micro- and nanoscale plastic contaminants is commonly based on colloidal coagulants (*20*). In our recent study, we introduced a class of effective microcleaning agents for capturing and aggregating MPs in water based on soft dendritic colloids (SDCs) (*21*, *22*). The SDCs have highly branched, fibrillar morphologies with extensive surface areas, enabling strong adhesion via van der Waals forces, similar to the adhesion mechanism of the nanofiber-lined gecko lizard feet (*21*). Constructed entirely from biopolymers such as chitosan and alginate, these microcleaners offer a sustainable and functionally versatile approach to MP removal. However, their effective capture range is generally limited to MPs on the micrometer scale.

The use of colloidal coagulants further faces the challenge of their efficient dispersal into large volumes of water for capturing the dispersed MPs. In one way of addressing these limitations, researchers have constructed self-propelling nano- and micromotors capable of navigating complex aqueous environments (*23*–*28*). These active particles can deliver remediation agents, adsorb contaminants, or enhance localized mixing to improve pollutant removal. However, many micromotor systems rely on catalytic reactions fueled by chemicals such as hydrogen peroxide (H_2_O_2_) or hydrazine (N_2_H_4_), which are neither environmentally friendly nor practical for open-water reservoirs (*24*, *25*). Alternative propulsion mechanisms based on surface tension or osmotic gradients have also been investigated (*22*). However, most nanomotor- and micromotor-based systems are optimized for particles smaller than 10 μm (*26*). These limitations leave a blind spot in current methods, which would struggle to efficiently capture MPs across the wide size range found in the environmental aquatic system. The polymer particulates found in natural bodies of water range from nanometer to centimeter size of very diverse polymer chemistry and degree of breakdown. The wide heterogeneity of MPs in terms of size, shape, density, chemical composition, and surface charge necessitates further development of environmentally safe MP cleaners operating in very broad range of sizes (*29*).

**Fig. 1.**
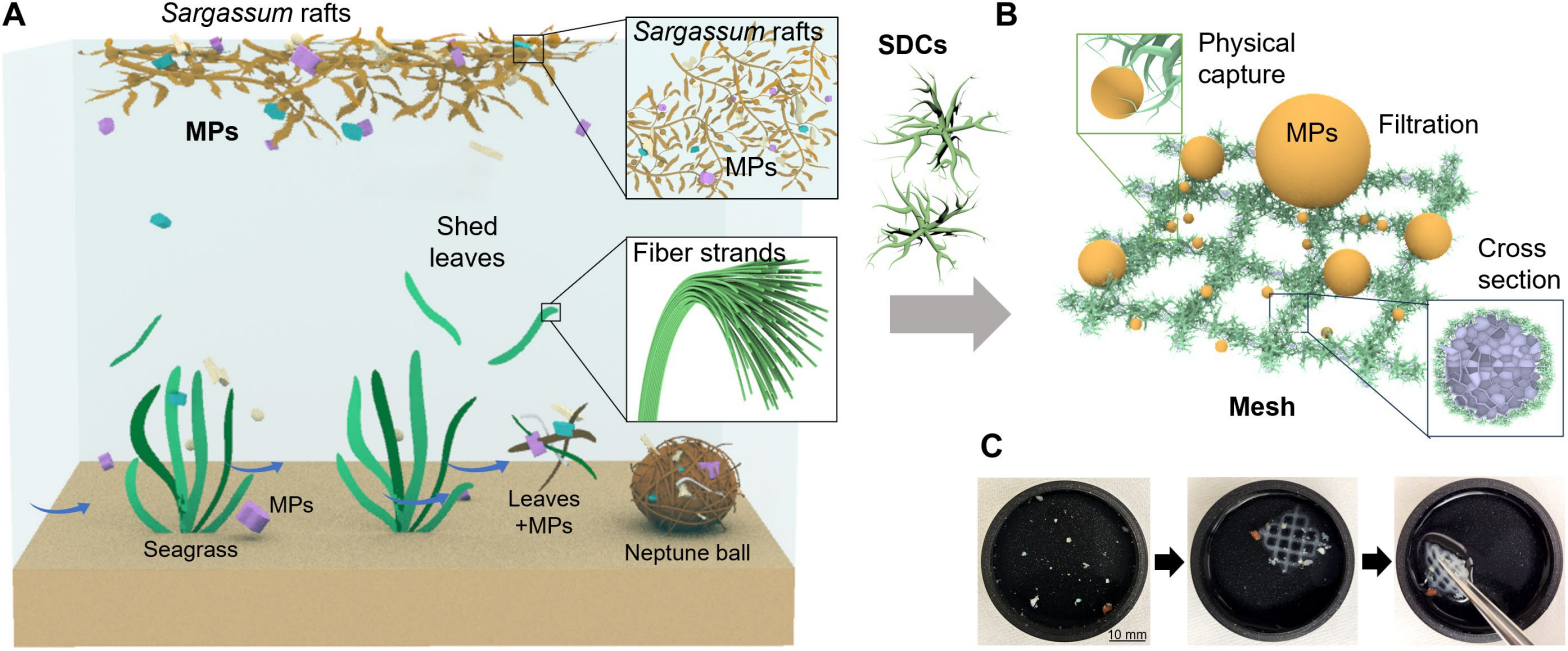
Overview of the biomimetic MP capture and removal system. (**A**) Illustration of the natural MP removal mechanisms found in *Sargassum* rafts and “Neptune balls” bundles from aggregated seagrass. (**B**) Concept of the hierarchically fibrillar mesh for MP capture inspired by these structures. (**C**) Example of mesh testing for MP removal.

In an alternative approach, nature has found a few efficient solutions for MP cleanup with the help of self-assembled structures formed by marine vegetation (Fig. 1A). Seaweeds and seagrasses exhibit highly branched, fibrous morphologies that enable them to entangle and capture polymer debris in aquatic environments. The floating pelagic *Sargassum* seaweed rafts, for instance, trap large amounts of buoyant plastic particles and other contaminants at the sea surface (*30*, *31*). Another type of natural MP-capturing seagrass structures is the so-called “Neptune balls,” formed from the fibrous remains of shed seagrass leaves. They capture the MPs in their porous networks. The Neptune balls then sink to the seafloor or wash ashore, enabling natural removal of the captured contaminants (*32*–*34*). These fascinating structures capture MPs species through a combination of three mechanisms: (i) The first is physical entanglement—branched or foliose forms (e.g., *Chondrus crispus* with dense intermediate branching) excel at trapping MPs within crevices, branches, or thallus folds (*31*, *35*, *36*). Increased biomass, length, and structural complexity boost the surface area for interaction, with denser branching retaining more MPs than sparse or broad forms (*37*). (ii) The second is adhesion via mucus or surface properties—many seaweeds produce sticky mucus that binds MPs, making dislodgement harder; while softer (lower in cellulose) thalli allow particle penetration for firmer hold. This is evident in higher retention on real soft algae compared to rigid mimics (*30*, *35*). (iii) The last is flow-mediated transport—water dynamics plays a crucial role by enabling settling during low-flow pauses, thus enhancing MPs capture in complex morphologies (*38*).

Here, we report a class of hierarchically fibrillar mesh materials emulating the natural mechanisms by which *Sargassum* rafts and Neptune balls capture broad-spectrum NPs and MPs from aquatic environments. The meshes have the ability to collect macroscale MPs and floating debris at the water surface, while their nanoscale fibrillar surfaces capture MPs and NPs by strong adhesion, thus replicating the Neptune balls’ capability for combined entanglement and adhesion (Fig. 1B). The performance of these hierarchical microcleaners was evaluated against model and real MPs with varying surface charges and chemical composition. We demonstrate that this architecture improves the mechanical stability and enables the capture of a very broad range of NPs and MPs (≈300 nm to several millimeters). The meshes also enable easy removal of all captured NPs and MPs (Fig. 1C).

## RESULTS

### Design and fabrication of hierarchically fibrillar balls and meshes using SDCs

The design of the biomimetic broad-range MP cleaners synergistically combines two components: mechanically robust filament meshes that provide structural support and facilitate the physical filtration of larger MPs and a highly fibrillar outer layer that binds smaller MPs and NPs through van der Waals and electrostatic interactions (Fig. 1B). The inner hydrogel core supports the overall mesh structure and maintains its integrity during handling and immersion. This core is surrounded by a network of chitosan-SDCs (CS-SDCs) whose branches strongly adsorb smaller particles via electrostatic and van der Waals interactions. Simultaneously, the architecture’s millimeter-sized openings capture larger particles through physical entanglement, mimicking the mechanisms found in floating seaweed rafts and Neptune balls.

**Fig. 2.**
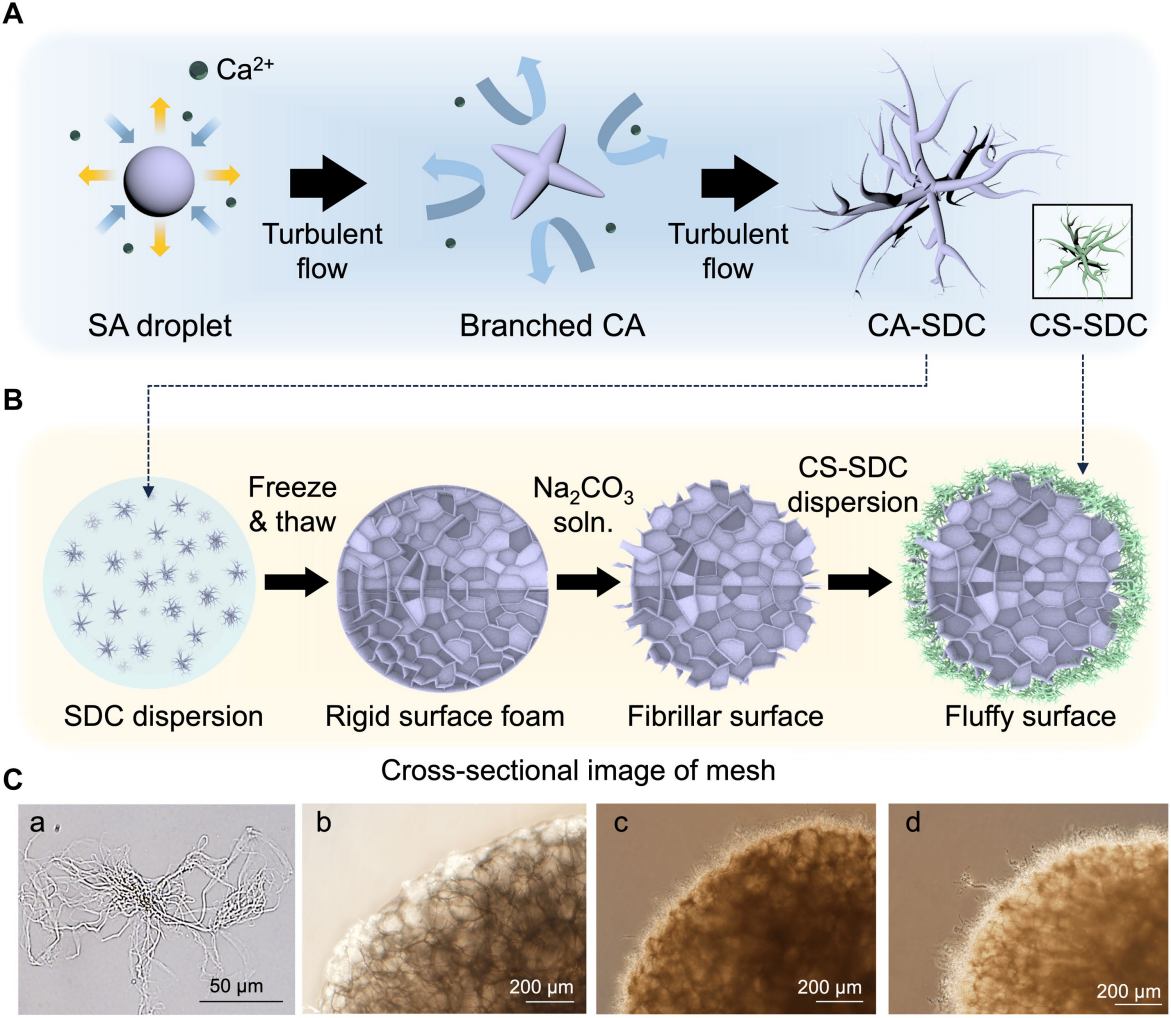
Fabrication and structure of hierarchically fibrillar balls and meshes using SDCs. (**A**) Schematic of the CA-SDC fabrication process via shear-driven precipitation and calcium ion cross-linking. (**B**) Cross-sectional perspective illustrating each step of the fabrication process for the hierarchical fibrous mesh using SDCs via the F-T method, carbonate treatment, and CS-SDC adsorption. (**C**) Optical microscopy of highly branched CA-SDC and cross-sectional microscopy images during the stages of the fabrication process illustrated in (B).

The fibrillar balls and meshes that we constructed are composed entirely of bio-derived materials: alginate and chitosan. Alginate is a natural polysaccharide extracted from brown algae, and chitosan is a seminatural polymer obtained by deacetylation of chitin from crustacean shells (*39*). These biopolymers are abundant, cost-effective, and sustainable, but they have to be reshaped and combined in mechanically robust morphologies (*40*, *41*). Thus, the first step was to convert them into SDCs that have a hierarchical, fibrillar morphology with high interconnectivity and large excluded volume by precipitating them into turbulently sheared medium (*42*, *43*). The SDCs could form a mechanically robust material network and serve as both the structural scaffold and the active adsorption sites for MP capture (Fig. 1B). Their multiscale branching enhances adhesion via van der Waals forces—an effect akin to the gecko leg’s adhesion mechanism—driven by the phenomenon of contact splitting (*42*). Contact splitting occurs when a fibrillar network divides a single contact area into thousands of nanoscale contact points. Similarly to a gecko’s foot lining, this vastly increases the surface area and increases the van der Waals forces, enabling, in this case, superadhesive trapping of micro- and nanoscale plastics (*44*–*46*). The alginate SDCs are synthesized by precipitating alginate through calcium ion-induced cross-linking in a turbulently sheared liquid medium (Fig. 2A). The Ca^2+^ ions promote interchain association at the junction zones formed by the G-blocks of alginate (fig. S1) (*41*, *47*). The resulting SDCs from calcium alginate (CA-SDCs) exhibit a corona-like structure in which fine nanofibrils spread out from a thicker backbone. The CS-SDCs were prepared by a procedure of ionic precipitation with citrate (*21*).

The thus-prepared CA- and CS-SDCs served as building units of adhesive porous balls and meshes with nanofibrillar surfaces that capture MPs. First, we fabricated robust hydrogel balls composed entirely of SDCs (Fig. 2, B and C). CA-SDC dispersion was shaped into spheres via three-dimensional printing or by molding with polydimethylsiloxane templates (fig. S2A). We then applied the freeze-thaw (F-T) method to induce physical cross-linking: Ice crystal formation concentrated the SDCs into distinct domains, promoting network formation through physical entanglement and interparticle cohesion, as analyzed in the next section. Following F-T gelation, the surface of the resulting porous hydrogel balls was treated with aqueous sodium carbonate (Na_2_CO_3_), which partially disrupted calcium cross-linking and produced a fibrillar outer layer (Fig. 2, B and Cc) (*48*). This so-called “fluffy” fibrillar layer served as an adhesive interface for subsequent coating. The balls were then immersed in CS-SDC dispersion, where the positively charged CS-SDCs adsorbed on their surfaces via electrostatic and van der Waals interactions and formed another branched “fluffy” overcoat layer (Fig. 2Cd). The resulting final structure is a hierarchically branched, double-layer composite made from bio-derived materials, with an inner porous CA-SDC hydrogel core for mechanical stability and an outer CS-SDC layer for NP and MP adsorption. This design enabled us to combine efficient particle capture and mechanical robustness.

While the fibrillar SDC balls effectively capture micro- and submicrometer particles through surface adhesion, their small surface/volume ratio and compact shape do not enable the capture of larger floating or suspended plastics. To overcome this limitation and enable scalable filtration and capture of a broader size range of MPs, we next transcribed the same SDC-based design into a mesh-shaped architecture. Accordingly, the CA-SDC dispersion was patterned into mesh form (fig. S2A) and coated with a “fluffy” outer layer following the same procedure used for the fibrillar balls, yielding a hierarchically fibrillar mesh. This open sticky mesh structure not only increases the available surface area for adsorption but also allows physical trapping of millimeter-scale MPs within the mesh openings. These structurally architected meshes are biomimetic models of natural *Sargassum* rafts, which trap and retain floating debris in marine environments through a combination of fibrous entanglement and multiscale porosity.

### Enhancing the mechanical entanglement of the SDCs via F-T cycling

**Fig. 3.**
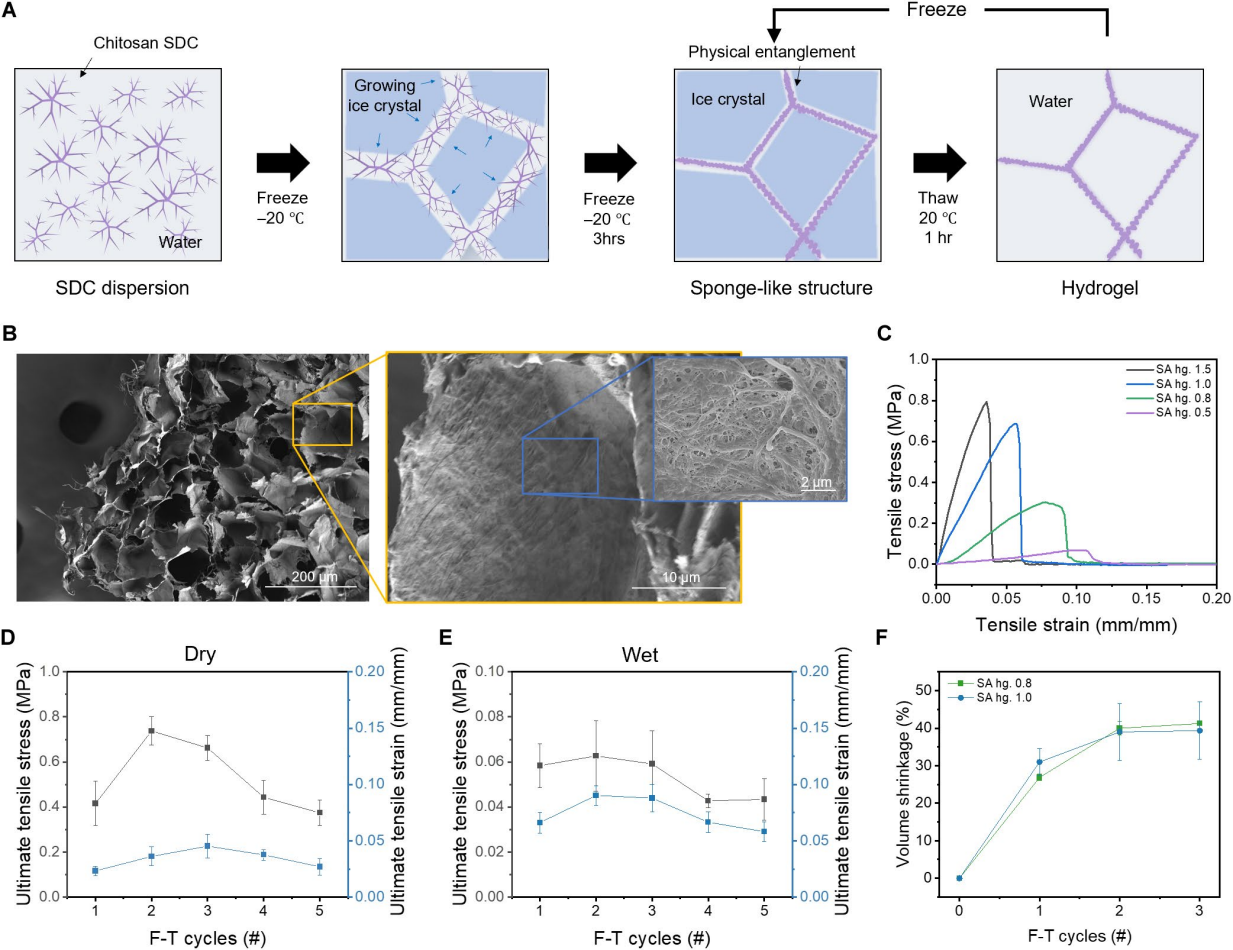
Mechanical reinforcement of SDC hydrogels through physical cross-linking induced by F-T cycles. (**A**) Illustration of F-T–induced gelation through confinement and aggregation of SDCs between growing ice crystals. hr, hour. (**B**) Cross-sectional SEM images of CA-SDC hydrogel after multiple F-T cycles. (**C**) Tensile stress-strain curves of CA-SDC hydrogels (hg) with different initial concentrations of SDC dispersion after repeated F-T cycles. (**D** and **E**) Dependence of the tensile stress and strain of CA-SDC hydrogels in the dry (D) and wet (E) states on the number of F-T cycles. (**F**) Volume shrinkage of CA-SDC hydrogel cores as a function of the number of F-T cycles.

The engineered assembly of the highly adhesive SDCs into larger-scale ball- and mesh-shaped architectures enables the capture of a much broader range of MPs and larger polymer fragments than possible with individually dispersed microcleaners. However, forming these standalone macroscopic structures requires bulk mechanical reinforcement. The highly fibrillar, “gecko leg” SDC morphology facilitates their assembly in mechanically stable meshes (Fig. 2B). To consolidate the percolated fibrillar network in the meshes, we applied F-T cycling, a simple and scalable postprocessing treatment (*49*). This process forces the SDCs into tight proximity through controlled cycles of freezing and melting, increasing the number of contacts between the fibrils and enhancing their physical binding (*50*). Consequently, this consolidates the highly adhesive SDCs into mechanically stable mesh filament cores at low polymer volume fractions (fig. S2B). Unlike the conventional freeze-drying process, which operates at ultralow temperatures (>−200°C), the F-T method is conducted at moderately low temperatures (≈−20°C) over extended freezing times (*51*). During freezing, water crystallizes and squeezes the SDCs into the narrow spaces between the growing ice crystals, concentrating them in confined regions (Fig. 3A). This treatment increases the number of contacts and enhances the network integrity. As shown in fig. S3, a more densely interconnected network was formed as a result of the F-T cycles. Upon thawing, the concentrated regions remained permanently cross-linked through hydrogen bonding and van der Waals forces, forming a robust three-dimensional network structure. This approach enables the fabrication of porous and mechanically robust gel cores of balls and mesh filaments. The process avoids chemical cross-linkers, making it environmentally friendly and suitable for large-scale fabrication.

The degree of network integration and cross-linking could be increased by repeated F-T cycling. The structural network evolution was characterized using scanning electron microscopy (SEM) (Fig. 3B). After three F-T cycles, CA-SDC hydrogels exhibited uniform, compact cross sections resembling honeycomb-like porous networks, with pore sizes typically <100 μm. The flat and interconnected pore walls are attributed to ice crystal growth, which compresses the fibrils into dense walls around the ice cores (*52*). As shown in the higher-magnification section of Fig. 3B, the SDCs compressed by the expanding ice crystals aggregated into foam-like sheet structures. While the F-T method was applicable to both CA-SDC and CS-SDC systems, only CA-SDCs formed rigid, sheet-like pore walls; CS-SDCs produced thicker, more heterogeneous walls composed of loosely entangled fibrils, having lower mechanical strength (figs. S4 and S5A).

The mechanical performance and pore structure were influenced by both the number of F-T cycles and the initial SDC concentration. Higher initial SDC concentrations resulted in thicker walls, yielding denser and mechanically stronger cores (Fig. 3C). As shown in Fig. 3 (D and E), increasing the number of F-T cycles improved the mechanical strength in both dry and hydrated states because of increased hydrogen bonding and fibrillar entanglement, leading to a mechanically reinforced network. However, applying more than three cycles resulted in reduced elasticity and increased brittleness, possibly due to excessive compression and cross-linking.

The F-T process was accompanied by some degree of volumetric shrinkage resulting from structural densification. As shown in Fig. 3F, the largest rate of shrinkage occurred during the first freezing cycle when the initial percolated fibrillar network was established. Subsequent F-T cycles produced incremental densification, with a saturation point reached after two to three cycles. This additional densification correlated with improved gel firmness and strength.

### Role of the hierarchically fibrillar mesh in the MP capture

**Fig. 4.**
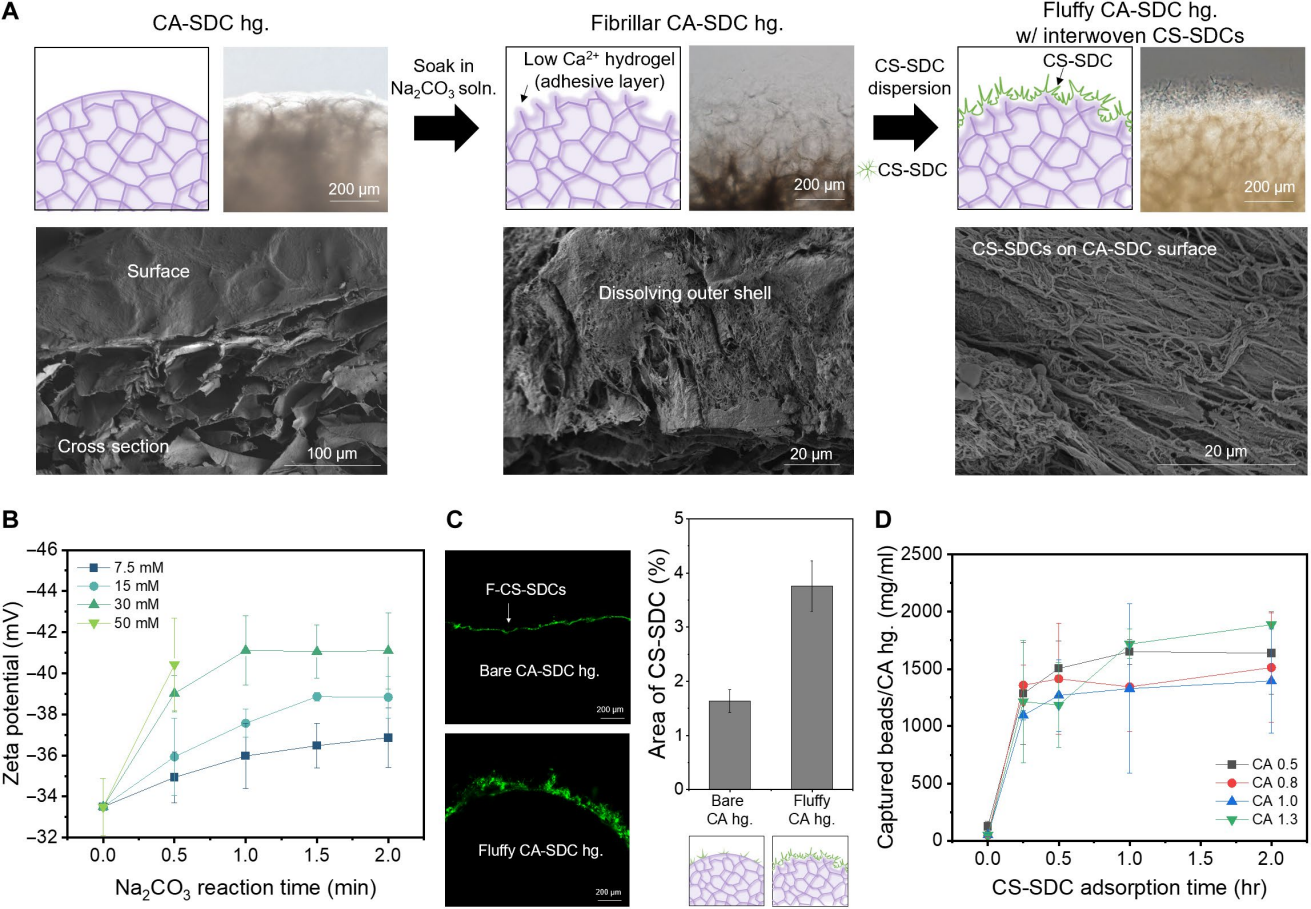
Surface modification of CA-SDC filament cores to coat them with a “fluffy” CS-SDC layer for enhanced MP adsorption. (**A**) Schematic of carbonate treatment and CS-SDC coating to fabricate a bilayer mesh structure. (**B**) Surface zeta potential of CA-SDC hydrogels as a function of Na_2_CO_3_ treatment time at varying concentrations. (**C**) Confocal microscopy images of bilayered CA-SDC hydrogels with CS-SDCs labeled with fluorescein isothiocyanate. (**D**) Comparison of MP capture efficiency across hydrogels of equal volume but different core CA-SDC.

The F-T process produces self-supporting gel filaments with cores composed of physically percolated SDC networks. However, during this process, the consolidation of the SDCs on their outer surfaces resulted in smooth, sheet-like surfaces enclosing the filaments (Fig. 4A). This smooth rigid surface reduces the exposure of the original fibrillar morphology, thereby suppressing the adhesivity of MP-capturing CS-SDC coatings. Therefore, we added a final surface modification step to “re-fibrillate” the outer layer, restoring the “fluffy,” adhesive SDC texture necessary for highly effective MP capture (Fig. 4A). To improve the mesh’s surface adhesivity, we developed a posttreatment process that modifies the CA-SDC core surface, creating an open fibrillated texture that facilitates the subsequent attachment of a CS-SDC overlayer. The final “fluffy” surface features a high density of CS-SDC fibrillar branches that are able to capture MPs and NPs through physical adsorption.

To re-fibrillate the condensed surface layer, the CA-SDC hydrogels were immersed in an aqueous solution of Na_2_CO_3_. Carbonate ions (CO_3_^2−^) chelate calcium ions (Ca^2+^) from the hydrogel matrix, forming calcium carbonate and thereby disrupting the surface cross-links (fig. S6). This treatment reexposes the internal branched SDC structures and restores a high-surface-area morphology. Optical microscopy revealed visibly rougher, more open surfaces, and SEM analysis confirmed partial dissolution of the compact shell, revealing the underlying fibrillar network (Fig. 4A, middle).

The reduction of Ca^2+^ ion concentration on the CA-SDC hydrogel surface promotes entanglement and facilitates interpenetration with the positively charged CS-SDCs via electrostatic interaction (*53*). Untreated CA hydrogels exhibit a low absolute zeta potential, likely due to partial charge neutralization of the polyelectrolyte network by Ca^2+^ (*54*). Treatment with Na_2_CO_3_ increases the magnitude of the zeta potential of the hydrogel (Fig. 4B), with greater increases observed at longer treatment times and higher Na_2_CO_3_ concentrations, up to a saturation point. Excessively high Na_2_CO_3_ concentrations (≥50 mM), however, rapidly degrade the hydrogel core. The moderately enhanced negative surface charge by carbonate treatment (fig. S7A) strengthens the electrostatic adhesion with positively charged CS-SDCs. The combination of a fibrillated CA-SDC hydrogel surface and increased surface charge markedly improves the adsorption of CS-SDC coatings (fig. S7B). Compared to flat-surface hydrogels, the fibrillated CA-SDC hydrogel cores demonstrated a thicker CS-SDC layer (Fig. 4C). These CS-SDC layer–coated fluffy meshes act as primary capture interfaces for MPs based on both electrostatic and van der Waals interactions.

We quantified the MP capture efficiency as a function of the CS-SDC adsorption time using CA-SDC hydrogel cores of varying initial polymer concentrations but constant volume. Polystyrene (PS) beads were used as model MPs. As shown in Fig. 4D, bead capture efficiency increased rapidly within the first 15 min of CS-SDC adsorption before plateauing, indicating saturation of the surface binding sites. Notably, the MP capture efficiency per unit hydrogel volume was consistent across samples, suggesting that the capture capacity is primarily governed by the accessible surface area, rather than bulk polymer mass.

**Fig. 5.**
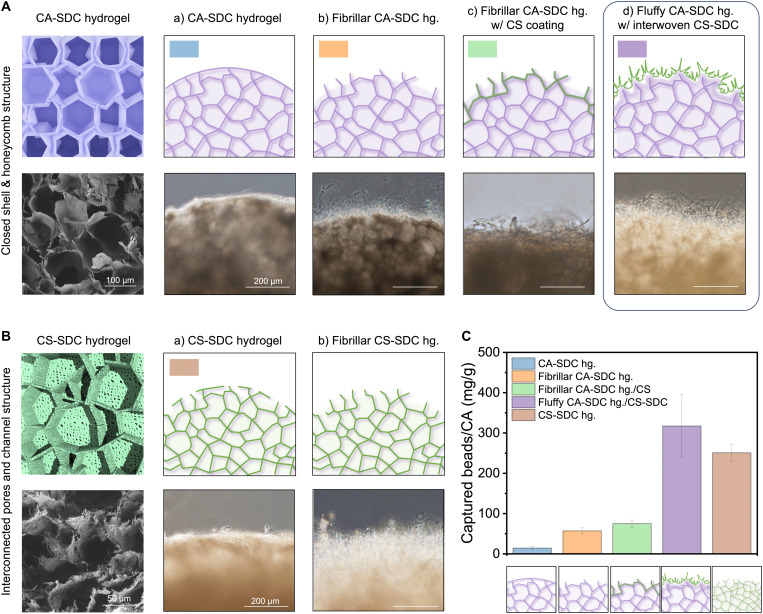
Morphological variation of filament cores fabricated via the F-T method followed by surface modification. (**A** and **B**) Porous gels made from alginate (A) and chitosan (B), showing differences in pores and surface structures. (Aa) Bare CA-SDC cores with a smooth, flat surface. (Ab) Carbonate-treated CA-SDC cores with a rough, fibrillar surface. (Ac) CS-treated CA-SDC porous hydrogel showing reduced surface texture but increased surface charge. (Ad) “Fluffy” CA-SDC filament interwoven with CS-SDCs displaying dense, fibrillar architecture. (Ba) Untreated CS-SDC gels with a porous yet relatively smooth surface. (Bb) CS-SDC core treated with an acetic acid solution, showing enhanced surface fibrillation. (**C**) Effect of base material and surface modification on MP capture efficiency. The highly fibrillated “fluffy” CA-SDC filaments coated with CS-SDCs show superior MP capture efficiency.

By combining F-T processing with carbonate treatment, we fabricated and compared the properties of four CA-SDC–based composite filaments and two CS-SDC–based filaments of distinct surface morphologies (Fig. 5). SEM analysis showed that the hydrogels contained large pores (∼100 μm) formed by ice crystal growth, but pore shapes and wall structures varied with material composition and the applied posttreatment procedures.

Among the CA-SDC–based hydrogel cores, untreated CA-SDC cores exhibited a smooth, compact surface (Fig. 5Aa), whereas carbonate-treated CA-SDC hydrogel cores had open, fibrillar surfaces resulting from partial removal of the densified outer layer (Fig. 5Ab). When the treated CA-SDC core was coated with chitosan, some of the nanofibrillar features coarsened, but the branched structure remained (Fig. 5Ac). The fibrillar CA-SDC hydrogel core interwoven with CS-SDCs exhibited the most pronounced surface morphology, with an extensive CS-SDC fibrillar network on the surface (Fig. 5Ad). For pure CS-SDC–based hydrogels, the untreated CS-SDC hydrogel core retained a fibrillar structure after F-T processing (Fig. 5Ba). Subsequent treatment with 0.5% acetic acid further enhanced the fibrillar texture (Fig. 5Bb). Unlike the CA-SDC hydrogel cores, which exhibit closed pores and dense walls, the CS-SDC mesh filaments have interconnected pores and porous walls. This open architecture leads to excessive hydrogel core swelling and therefore renders the core more fragile (Fig. 5Bb).

The efficiency of MP adsorption was evaluated at a fixed initial PS bead concentration and equal total mass of biopolymer present for all hydrogel types (Fig. 5C). Bead capture efficiency increased with the degree of chitosan or CS-SDC surface treatment, attributable to a change in their zeta potential: The initially negative CA-SDC hydrogel surfaces became more positively charged after chitosan treatment, increasing the electrostatic attraction with negatively charged PS beads (figs. S5B and S7B). The hydrogels with “fluffy” surface morphologies consistently outperformed flat-surfaced variants, underscoring the importance of high surface area and branched overlayers for maximizing MP contact. The highest bead capture efficiency was observed with the fluffy CA-SDC hydrogel interwoven with CS-SDCs, demonstrating a synergistic effect of morphological and electrostatic optimization.

### Performance of the mesh cleaners in MP capture

Our hypothesis, derived from the functionality of natural seaweed aggregates, was that hierarchical mesh microcleaners can capture a wide diversity of MPs in terms of size, chemical composition, and interfacial properties. We first evaluated the broad-spectrum capture capabilities of the “fluffy” meshes by using a range of model MPs as test systems. The model particles differed in size, surface charge, and polymer composition. For each experiment, the microsphere dispersion (with initial parameters provided in tables S1 and S2) was gently agitated in a dish while adding the mesh (made of 5 mg of CA-SDC). The mesh has a highly porous architecture with a substantially low measured density of 0.0535 g/cm^3^, enabling it to remain persistently buoyant, likely due to air pockets trapped in its pores (fig. S8). The microsphere concentrations before and after the capturing process were quantified using either light absorbance or fluorescence intensity measurements, and removal efficiencies were calculated following previously reported protocols (*21*, *22*). A schematic of the quantification procedure is provided in fig. S9.

**Fig. 6.**
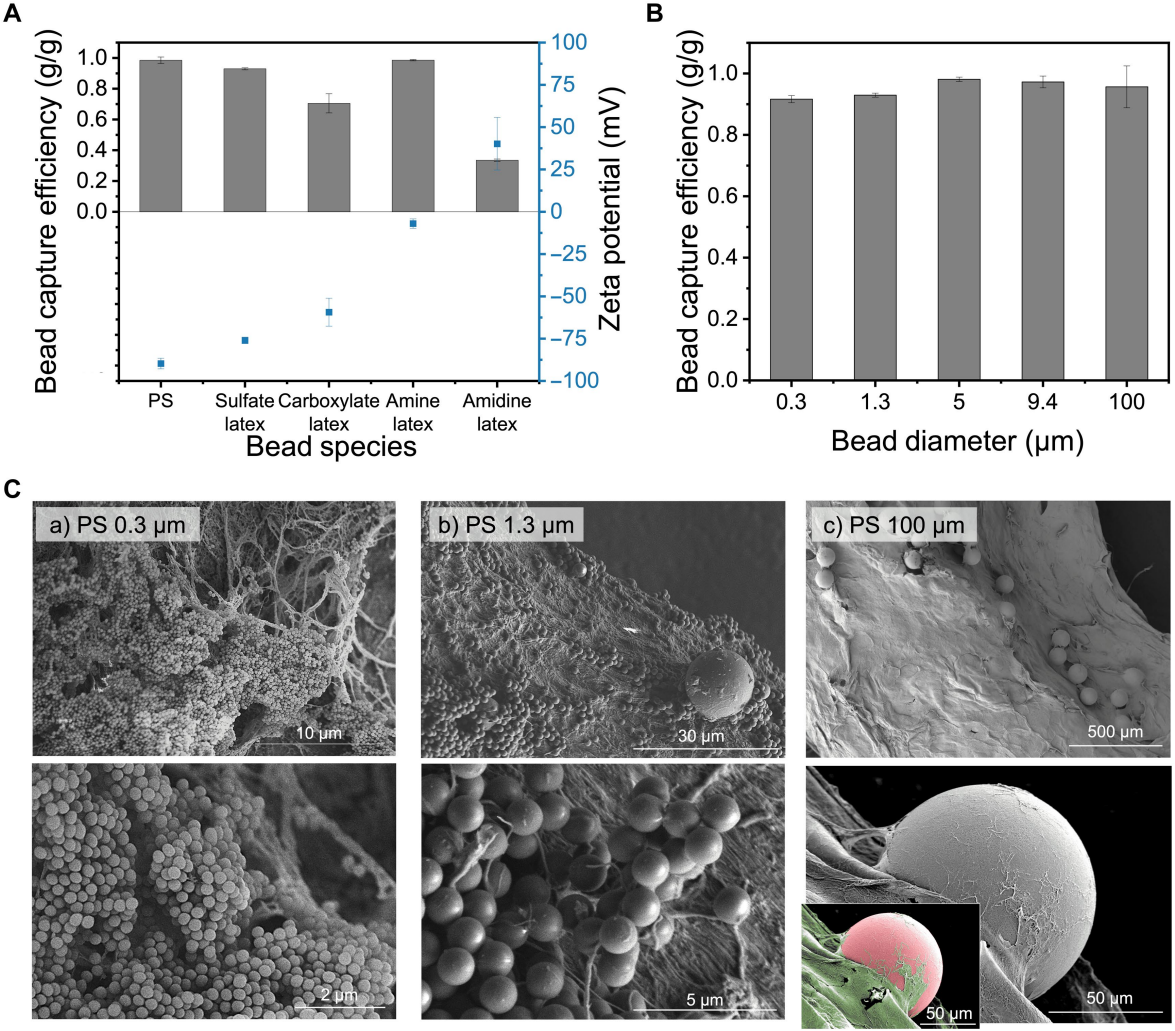
Examples of the multiscale NMP capture efficiency of the “fluffy” mesh. (**A**) Removal efficiencies of PS MPs with different surface functional groups, resulting in varying zeta potential. (**B**) Removal efficiencies of PS NPs and MPs with sizes ranging from 300 nm to 100 μm. (**C**) SEM images of PS NPs and MPs captured on the mesh: 300 nm (left), 10 μm (middle), and 100 μm (right). The artificially colored SEM image of a 100-μm PS MP (inset) shows that particles are attached through physical adhesion at numerous contacts with the fibrillar surface.

For the experimental results shown in Fig. 6A, all model MPs were composed of latex microbeads with similar diameters (0.97 to 1.3 μm) but with different surface functional groups, resulting in negative, neutral, or positive surface charges. The model particles’ properties are summarized in detail in table S1. The capture efficiency varied with the surface charge. As expected, negatively charged PS microspheres, including bare and sulfate-functionalized latex beads, exhibited the highest removal efficiencies because of strong electrostatic attraction with the positively charged mesh surface, achieving values of 98.5 and 93.0%, respectively. Amidine-functionalized latex beads, which are positively charged, showed a lower but still substantial removal efficiency of 33.5%, in good correlation to our previous experimental and Derjaguin–Landau–Verwey–Overbeek model studies of SDC-particle interactions that concluded that both electrostatic forces and van der Waals interactions contribute to the adsorption and adhesion mechanisms (*21*, *22*). The generally weaker, but omnipresent, van der Walls forces are amplified by the massive surface area provided by the fluffy mesh surface through the mechanism of contact splitting within the branched nanofibrillar network (*42*).

The influence of particle size on the mesh removal performance was assessed using PS particles ranging from 300 nm to 100 μm in diameter (Fig. 6B). The concentration used in these experiments (0.1 mg ml^−1^) was considerably higher than commonly environmentally encountered levels (*55*). Under these conditions, the number of beads per milliliter varied markedly—from 6.7 × 10^9^ ml^−1^ for 300-nm particles to 1.8 × 10^2^ ml^−1^ for 100-μm particles (table S2). Despite this large variation, all mesh systems achieved similar removal efficiencies, averaging above 90 wt % across all particle sizes. SEM imaging (Fig. 6C) confirmed that during model NP and MP capture by the fibrillar mesh, there is little effect of bead size on the overall removal performance. One interesting detail revealed by the images is that smaller beads frequently formed multilayer assemblies on the mesh surface (Fig. 6Ca), enabled by the branched fibrils extending away from their surface. A similar stacking effect was observed for 1.3-μm (fig. S10A) and 10-μm beads (Fig. 6Cb), where particles became entangled within the fibrils. Additional images show how numerous microbeads stacked onto SDC fibrils to form larger clusters (fig. S10B). Even for 100-μm particles, the mesh achieved a high removal efficiency of 95.7 wt %, highlighting its ability to capture both small and large plastic particles through multiple contact points, analogous to gecko-inspired adhesion (Fig. 6Cc). Furthermore, the mesh (of 5-mg solid content) demonstrated highly concentration-independent capture efficiency performance, maintaining a robust removal efficiency of >95% across initial 1.3-μm bead concentrations ranging from 4.14 × 10^6^ to 4.14 × 10^8^ beads/ml (fig. S11).

**Fig. 7.**
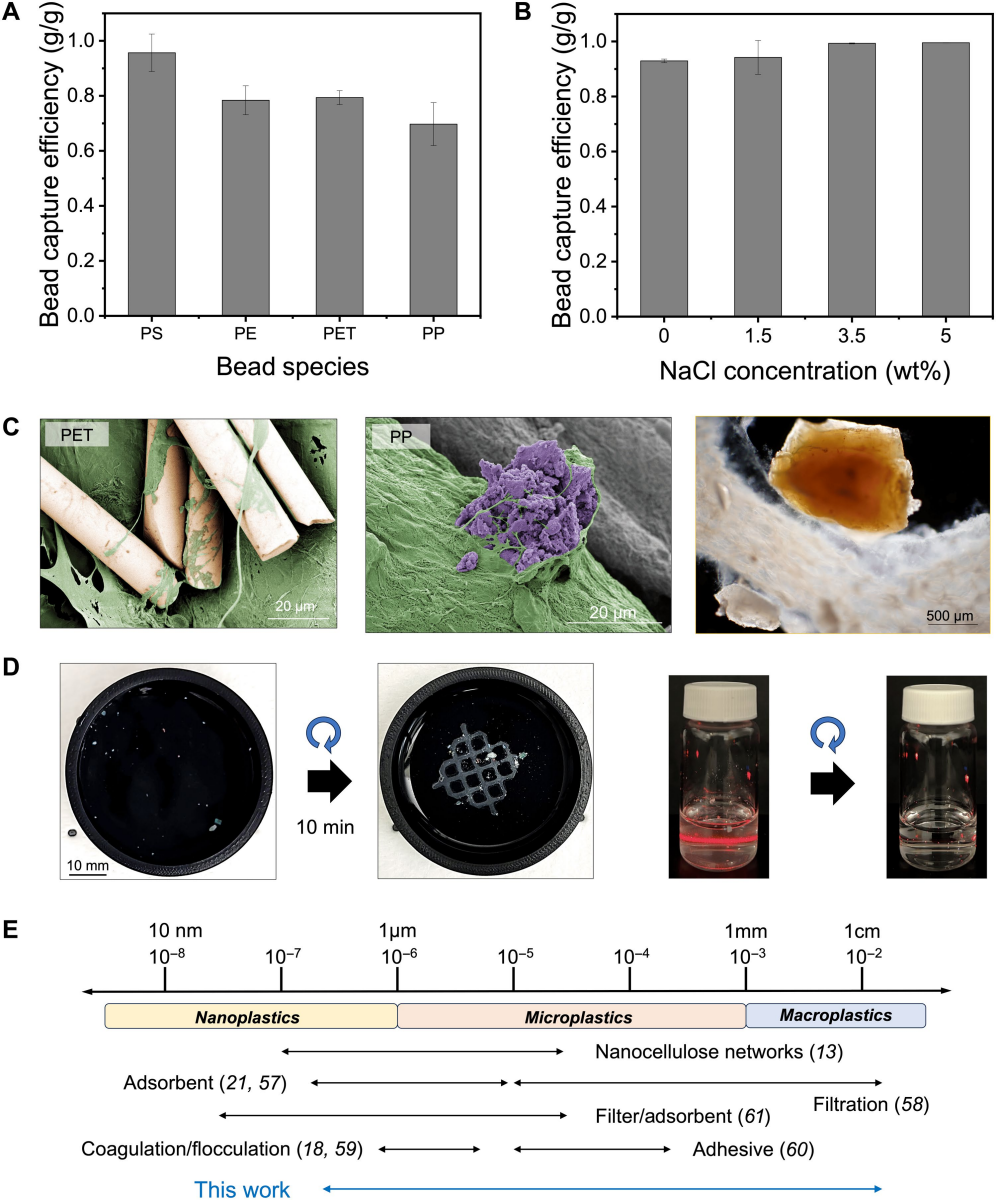
Capture performance of the mesh for MPs of different polymer and medium compositions and morphologies. (**A**) Removal efficiencies of model MPs from different polymers, including PS, PE, PET, and PP. (**B**) Efficiencies of PS microsphere removal from water media of varying salinity. The capture efficiency remains high even at concentrations exceeding typical seawater levels (>3.5% NaCl). Initial concentration: 4.14 × 10^8^ beads/ml; mesh solid contents: 5 mg. (**C**) SEM images of PP and PET (bottom) MPs captured on the mesh surface. The colored images highlight the retention mechanisms through physical interception and numerous fibrillar contacts. The optical micrograph on the right illustrates the capture of ocean-derived large particles. (**D**) Capture of MPs collected from Kamilo Beach, Hawaii. Despite their heterogeneous sizes, shapes, and chemical compositions, the MPs were effectively entrapped by the mesh within 10 min. The tumbled bottled samples are transversed by a laser beam, demonstrating that the microscale (light-scattering) particles have also been removed after the mesh treatment. (**E**) Comparison of the size ranges of particles that can be captured by the micromesh cleaners and literature examples of other methods (*13*, *18*, *21*, *57*–*61*).

The MP capture performance was evaluated further using samples of plastic particles with different polymer compositions and morphologies (Fig. 7). The selected model polymers represent frequently detected types of MPs in aquatic environments (*16*), including PS, polyethylene (PE), polyethylene terephthalate (PET), and polypropylene (PP). Their physical properties are summarized in table S3. Most MPs in natural aquifers carry a net negative surface charge, typically arising from the use of anionic initiators or surfactants during polymerization, as well as adsorption of natural organic matter containing carboxyl and phenolic hydroxyl groups (*56*). Thus, we expected that the tested MPs will be captured with high efficiency by the positively charged meshes. The results in Fig. 7A confirm this hypothesis (exemplified in movie S1). However, PE, PET, and PP MPs displayed slightly lower removal efficiencies compared with PS MPs. This difference may be attributed to variations in the buoyancy and hydrophobicity of the model particles. PE and PP, with densities lower than that of water, tend to float at the air-water interface rather than remain suspended, reducing their collision frequency with a submerged mesh. Their hydrophobic surfaces also limit adhesion interactions with the hydrophilic or charged fibrillar surface of the mesh. PET particles, with higher densities, are more likely to sediment. In contrast, PS particles with densities close to the one of water and moderate surface wettability remain well dispersed, leading to more frequent collisions and adhesion to the mesh, which explains why they are captured at a comparatively higher efficiency.

To evaluate the meshes’ functionality in marine environments, where a high ionic strength can potentially interfere with electrostatic interactions, we conducted experiments assessing their stability and performance in saline water. Beyond static tensile strength, the environmental robustness of the meshes was confirmed through vigorous agitation test (80-rpm orbital shaking), modeling extreme environmental shear. The meshes exhibited minimal mass loss in both deionized (DI) and saline [3.5 wt % sodium chloride (NaCl)] water over 24 hours, demonstrating good structural stability when subjected to hydrodynamic shear forces and ionic exchange (fig. S12).

Alongside structure integrity, we evaluated the MP capture efficiency in solutions of varying NaCl concentrations. While high salinity screens electrostatic interactions, which might be expected to hinder capture, we observed that salinity did not affect the overall performance to a large extent. The increased ionic concentrations only slightly enhanced the adsorption of the PS beads (Fig. 7B). This occurs because the high ionic strength effectively screens the electrostatic repulsion between the negatively charged PS beads themselves, allowing them to aggregate and densely adsorb onto the high-surface-area mesh. Consequently, across a broad range of NaCl concentrations, including those exceeding typical seawater levels (>3.5 wt % NaCl), the capture efficiency remained robustly high. Together, these findings suggest that ball-shaped and mesh cleaners can function effectively and maintain stability in harsh, high-salinity marine environments.

As shown in Fig. 7C, the tested MPs also exhibited different shapes and size distributions. Regardless of these variations, all were effectively retained by the mesh through physical interception, aided by the fibrillar overcoat that promotes multipoint contact and adhesion (Fig. 7C). Low-magnification SEM images (fig. S13) further show MPs distributed across the mesh surfaces, while the cross-sectional confocal image (fig. S14) reveals how particles of various sizes and shapes were captured within the CS-SDC layer by getting entangled within the fibrillar network. Together, these results demonstrate the universal NP- and MP-capturing capacity of the mesh regardless of the surface charge, size, shape, or polymer type of the target particles.

To further evaluate the practical applicability of such microcleaners, we tested the mesh performance with MP samples collected from Kamilo Beach, Hawaii, provided by the Plastic Ocean Project. To initially characterize the highly heterogeneous environmental samples, we analyzed the size distribution of both the floating and suspended MP fractions. This analysis revealed that most of the dispersed submicrometer plastics fell within the 200- to 300-nm range (fig. S15A), while the macroscopic floating particles exhibited an exceptionally broad size distribution, with fragments spanning from ∼100 μm to more than 5 mm (fig. S15B). These highly heterogeneous MPs, encompassing a diverse range of sizes and chemical compositions, were then suspended in water (0.2 wt % MP solid in 6 ml) and treated with a fluffy mesh (5-mg solid content). As shown in Fig. 7D, the mesh effectively entrapped both micrometer- and millimeter-scale particles of different polymer types. Optical images revealed that millimeter-scale particles were captured within 10 min, while smaller particles were largely removed within 24 hours. The overall removal efficiency for these real-world MPs in a tumbled vessel (Fig. 7D, left) reached 92% (5-mg mesh captures 11 mg of MPs), demonstrating the mesh’s capability to capture plastic particulates across a wide size and material spectrum (movie S2). These results indicate that the SDC-based meshes are not limited to the capture of model particles but also perform efficiently with samples of diverse origin and composition, underscoring their potential for future remediation applications.

## DISCUSSION

We demonstrate that hierarchically fibrillar hydrogel meshes, constructed entirely from architected SDCs of alginate and chitosan, provide an effective and sustainable approach for capturing MP particles across a wide spectrum of sizes, surface chemistries, and morphologies. The multiscale architecture mimics biological adhesion and particle entrapment processes—such as the ones that allow for natural structures from seaweed and seagrass to collect MPs from seawater (*56*). By combining a mechanically robust inner core framework with an outer “fluffy” fibrillar layer consisting of nanoscale branches, the mesh leverages dual mechanisms of physical sieving of larger particles and adhesive adsorption of MPs and NPs. The ability of the meshes to consistently capture and remove a broad range of plastic particles is realized by the engineered sheath of hierarchically branched fibers around the mesh filaments. The “fluffy” meshes were proven to be effective in collecting a multitude of polymer particles with different compositions and shapes commonly found in aquatic systems, including PS, PE, PP, and PET. Capture efficiencies of >90% were achieved for negatively charged and neutral MPs of sizes spanning from the nanoscale (300 nm) to microscale (100 μm), confirming the synergetic effect of fibrillar adhesion and structural entanglement. Even positively charged particles were removed at an efficiency exceeding 30%, supporting the finding from our previous study (*21*) that the universal capture capability of the “gecko leg” fibrillar surfaces stems from the joint effects of contact splitting and van der Waals attraction that change little with the polymer charge and medium composition. These results establish the SDC-based mesh as an example of a biomimetic platform capable of broad-spectrum plastic remediation.

Similar to the *Sargassum* rafts and Neptune balls that serve as inspiration for this study, our architected meshes are fully biodegradable and environmentally compatible. The successful capture of heterogeneous, ocean-extracted MPs demonstrates their effectiveness under environmentally relevant conditions, reinforcing its potential for real-world remediation applications. Beyond their high capture efficiency, the multistage fabrication strategy of the biomimetic meshes addresses two persistent challenges in biopolymer-based materials: mechanical fragility and scalability of highly porous particle networks. The F-T processing of samples in simple molds densified the hydrogel network, improving robustness without chemical cross-linkers, while carbonate treatment restored accessible surface fibrils to support efficient CS-SDC adsorption. This scalable, solvent-free postprocessing pathway enhances both material performance and environmental safety, underscoring its suitability for practical application. The meshes capture both millimeter-sized and larger particles in their network, while micrometer-sized and submicrometer MPs are captured by the “fluffy” fibrillar surfaces. A tentative comparison of the size range that can be captured by these structures and various MP collection strategies described in the literature (*13*, *18*, *21*, *57**–**60*) is presented in Fig. 7E, demonstrating the meshes’ ability to simultaneously capture a very broad spectrum of MPs, from submicrometer particles up to millimeter-scale fragments. Furthermore, the minimal mass of environmentally safe biopolymers used in the meshes enables efficient large-scale collection of the collected MP mass together with the mesh cleaners. The collected MPs could be removed and reprocessed together with the inexpensive meshes that captured them, likely by microbial digestion and biosynthesis of more biopolymer for meshes. However, these concepts could be implemented in other formats too, such as coating more permanent types of meshes and sieves with adhesive layers of fibrillated biopolymers in SDC or similar morphology, thus capturing MP and NP particles in addition to larger polymer and other debris.

Further advances in the development of biomimetic cleaners could be achieved by addressing several present limitations in future research. This study was conducted under controlled laboratory conditions, with MP concentrations considerably higher than those typically found in natural waters. The widely changing environmental variables in real-world aquifers such as salinity, the presence of natural organic matter, and biofilm formation may affect both adsorption kinetics and long-term mesh stability. While capture performance was presently demonstrated by benchtop device swirling of MP dispersions, the real mesh system may require an external driving force to effectively encounter MPs in natural aquatic environments and ensure high recovery efficiency. One way to accelerate the multiscale capture and remediation efficiency in large-scale complex aquatic systems could be to combine the ball and mesh adhesive cleaners with active dispersion methods, such as the self-propelling and self-dispersing microcleaners that we developed earlier (*22*).

Overall, this study establishes the potential of hierarchically fibrillar, biopolymer-based meshes as an effective and environmentally responsible platform for broad-spectrum plastic particle capture and remediation. These biomimetic structures are made from completely sustainable materials via processes that are easily scalable and generally safe. Time will show whether this microcleaner type could become a common means for mitigating the growing challenge of MP pollution in aquatic systems, where they could supplement the structurally analogous natural seagrass mats that already perform similar functions.

## MATERIALS AND METHODS

### Liquid-shear fabrication of biodegradable SDCs

The SDCs were fabricated using our previously reported liquid shear precipitation method (*21*, *22*, *42*, *43*). To make CA-SDCs, alginic acid sodium salt (SA; 120 to 190 kDa, Sigma-Aldrich) was dissolved in water at 4 wt %. A colloidal high-shear mixer (IKA Magic Lab, IKA Works Inc., US) was filled with 200 ml of 6.75 mM calcium chloride solution (Sigma-Aldrich) as the precipitation medium. A 3-ml aliquot of the SA solution was injected directly into the shear zone of the device at 20,000 rpm using a machined injection attachment. The CA-SDCs formed instantaneously via ionic precipitation and remained suspended in the medium. For CS-SDCs, chitosan (low weight-average molecular weight, Sigma-Aldrich) was dissolved in a 1.5% acetic acid (Sigma-Aldrich) solution at 3 wt %. Three milliliters of the solution was injected into the shear zone of an IKA Magic Lab operating at 20,000 rpm, with 200 ml of 25 mM trisodium citrate dihydrate (NaCit, Thermo Fisher Scientific) as the precipitation medium. The CS-SDCs were ionically cross-linked with NaCit. After collection, both CA-SDCs and CS-SDCs were washed multiple times with water and stored either in water at 3°C. SDC suspensions were concentrated by centrifugation for 4 min at 3500 rpm and diluted to the desired concentration. Dry weight percentages were determined gravimetrically.

### Preparation of SDC hydrogels and double-layered SDC hydrogels

CA-SDC dispersions at 0.5, 0.8, 1.0, and 1.3 wt % were prepared in water. For F-T gelation, 2 ml of SDC dispersion was transferred to a polydimethylsiloxane mold with a mesh pattern and stored at −20°C for 6 hours, followed by 4 hours at 3°C (one F-T cycle). This process was repeated for one to three cycles. The surface modification leading to a “fluffy” texture was performed by immersing the gels in Na_2_CO_3_ solutions (7.5 to 50 mM) for 30 to 120 s with gentle swirling, followed by immediate rinsing with water three times. For fabricating the CS-SDC layer on the outer surface, the fluffy CA-SDC hydrogels were immersed in 0.1 wt % CS-SDC dispersion in water for 15 min to 2 hours, swirled to promote deposition, then transferred to water, and swirled for 30 min to remove loosely bound CS-SDCs before a final rinse.

### Characterization of SDCs, SDC hydrogels, and SDC cryogels

The volume of cylindrical hydrogels was determined by hexane displacement, and the degree of shrinkage was calculated from the change in volume before and after the F-T process according to the following equation: Shrinkage (%) = (*V*_i_ − *V*_f_)/*V*_i_ × 100, where *V*_i_ and *V*_f_ are sample volumes before and after the step, respectively (*13*). Bright-field optical microscopy and fluorescence optical microscopy (Olympus BX-61, Japan) were performed to observe dendritic particles and hydrogel surface architectures. Confocal scanning laser microscopy (Leica SP8, Germany) was performed to visualize the double-layered architecture of hydrogels. Field-emission SEM (SU8700, Hitachi, Japan) was performed to image SDC fibers and surfaces and the internal structures of hydrogels. Zeta potential measurements were performed using a Nano zetasizer (Zetasizer Nano ZSP, Malvern Instruments, UK) with a dip cell (ZEN1002, 2-mm electrode gap) in a 1 mM NaCl solution. The tensile properties of the gels were determined using a common testing machine (Instron 5943, US) with samples of 1.5 mm in thickness, 7 mm in width, 15 mm in length, and a 10 mm min^−1^ crosshead speed.

### Preparation of MPs

Microbead dispersions of varying concentrations were prepared by diluting a stock suspension of latex microspheres. Latex beads with different types of surface functionalization were purchased from various vendors: nonmodified latex (0.989 μm, Polysciences Inc.), sulfate latex (1.3 μm, Invitrogen), carboxylate latex (1 μm, Sigma-Aldrich), amine latex (1 μm, Sigma-Aldrich), and amidine latex (0.97 μm, Invitrogen). Latex beads of different sizes were also obtained as follows: latex (0.3 μm, Duke Scientific Corporation; 100 μm, Alpha Nanotech Inc.) and sulfate latex microspheres (1.4, 5, and 10 μm, Invitrogen). All working dispersions were prepared on demand by diluting these stock suspensions with DI water, except the saline medium experiments reported in Fig. 7B.

PE, PET, and PP microparticles were prepared via a precipitation process. PE (Sigma-Aldrich) was dissolved in toluene (Thermo Fisher Scientific) at 2 wt % at 130°C under stirring, while polyvinyl alcohol (PVA; Sigma-Aldrich) was dissolved in water at 2 wt % at 80°C. A 5-ml aliquot of the PE solution was poured into 50 ml of the PVA solution under stirring at 500 rpm, and the emulsion was gradually cooled to room temperature. The resulting microparticles were collected by filtration (Whatman filter paper), washed sequentially with acetone and water, and dispersed in water. For making PP microparticles, PP (Sigma-Aldrich) was dissolved in xylene (Sigma-Aldrich) at 4 wt % at 140°C under stirring, and a 3-ml aliquot of the PP solution was poured into 50 ml of 2 wt % PVA solution. The mixture was stirred at 300 rpm at room temperature for 24 hours, diluted 10-fold with DI water, and filtered, and the collected particles were dispersed in water. To make PET microparticles, PET (Sigma-Aldrich) was dissolved in a trifluoroacetic acid solution at 2 wt % at 40°C under stirring for 24 hours, diluted 10-fold with DI water, and filtered through a 0.1-mm mesh (Labalpha), and the collected microparticles were dispersed in DI water.

### Microbead capture tests

CA-SDC meshes were immersed in microbead dispersions and gently swirled for 24 hours to facilitate MP capture. A 200-μl aliquot of the dispersion was collected and analyzed for light absorbance or fluorescence intensity using a microplate reader (Synergy MX multimode microplate reader, BioTek, US). The concentration of unbound microbeads was determined using the Beer-Lambert law, *A* = ε × *l* × *c*_sample_, where *A* is the absorbance, ε is the absorptivity of the beads, *l* is the optical path length, and *c*_sample_ is the concentration of free beads in the sample. Calibration curves were generated from absorbance measurements of known microbead concentrations. Bead capture efficiency was calculated as eff_bead capture_ = (*c*_Initial beads_ − *c*_Free beads_)/*c*_Initial beads_. Further experimental details are available in our previous reports (*21*, *22*).
