# Supplementary material for: Artificial Neptune balls: Superadhesive biomimetic networks for broad-size microplastics capture and removal
Source: Sci Adv. 2026 Aug 5;12(32):eaeg0819. doi: 10.1126/sciadv.aeg0819 (PMC13440390; doi:10.1126/sciadv.aeg0819)
Supplement: Supplementary file 1 [file sciadv.aeg0819_sm.pdf]

Science Advances
AAAS

Supplementary Materials for

Artificial Neptune balls: Superadhesive biomimetic networks for broad-size microplastics capture and removal

Haeleen Hong *et al.*

Corresponding author: Orlin D. Velev, odvelev@ncsu.edu

Sci. Adv. **12**, eaeg0819 (2026)
DOI: 10.1126/sciadv.aeg0819

The PDF file includes:

Figs. S1 to S15
Supplementary Text
Tables S1 to S3
Legends for movies S1 and S2

Other Supplementary Material for this manuscript includes the following:

Movies S1 and S2

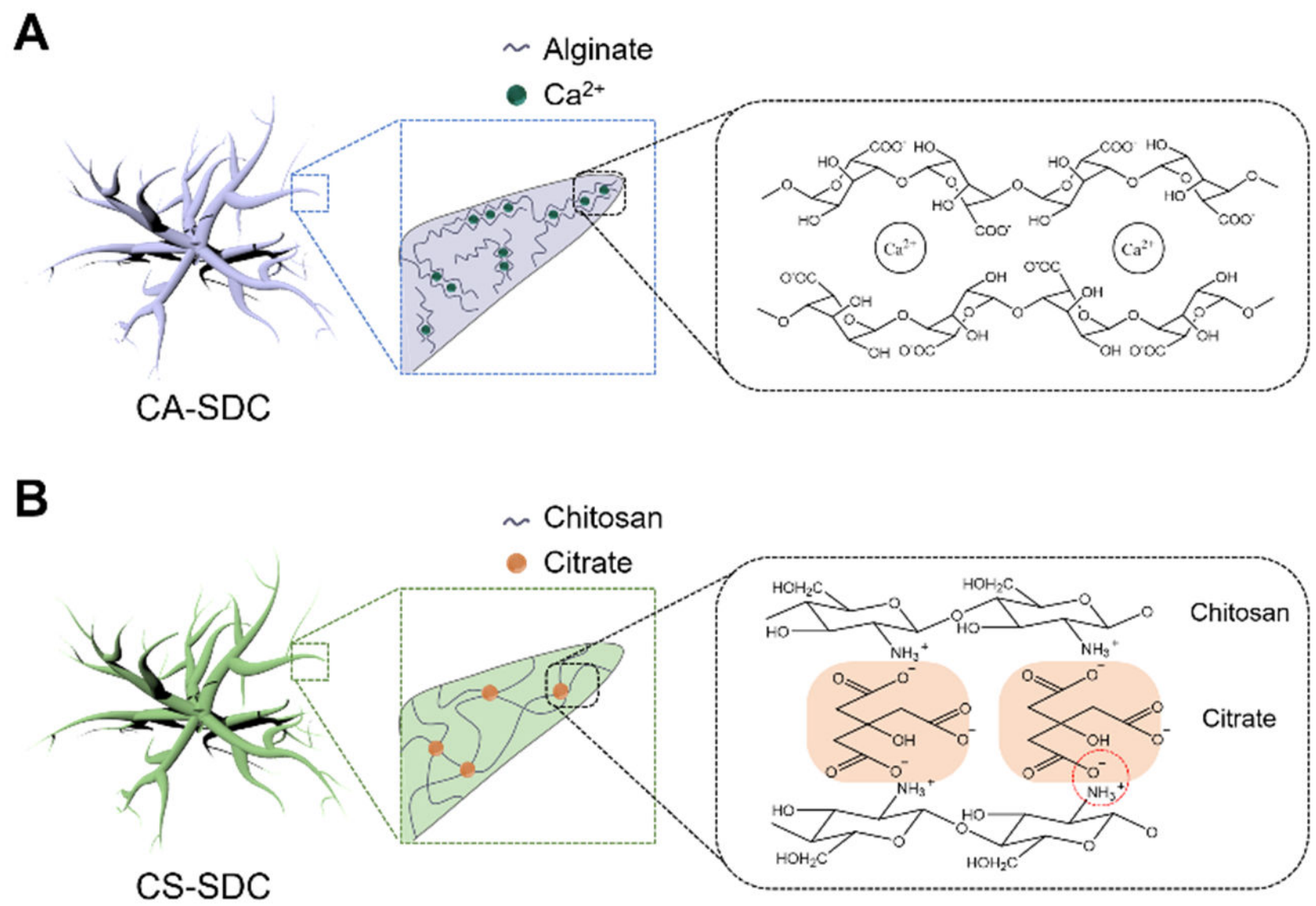

Fig. S1. Schematic illustration of the formation of soft dendritic colloid (SDC). (**A**) Calcium alginate-SDCs (CA-SDCs) are precipitated via ionic crosslinking between alginate and calcium ions, resulting in "egg-box junctions." (**B**) Chitosan-SDCs (CS-SDCs) formed through ionic crosslinking between chitosan and citrate ions.

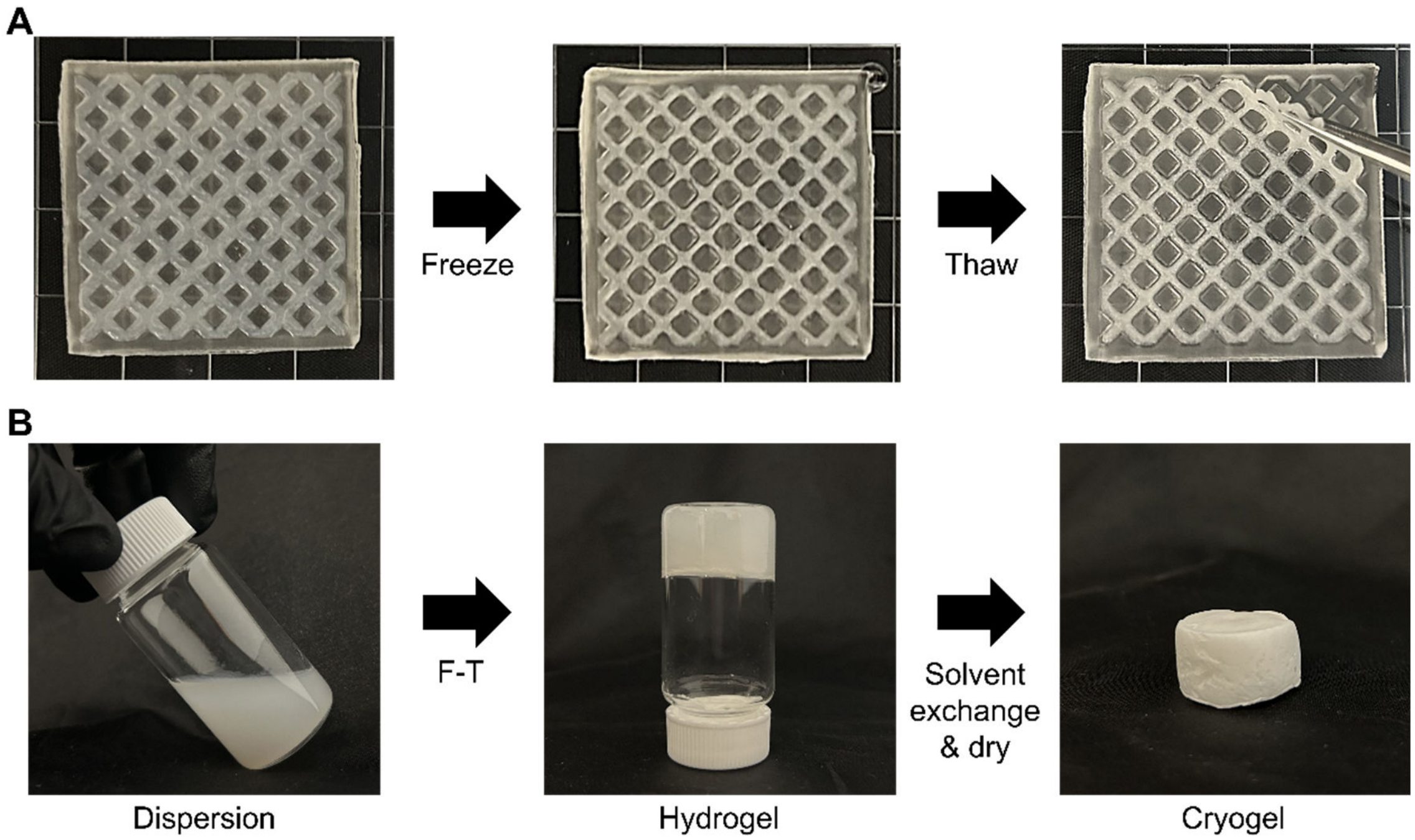

Fig. S2. Fabrication and morphology of the CA-SDC hydrogel core. (**A**) Photographs illustrating CA-SDC core via freeze-thaw (F-T) treatment to form the mesh shape. Initial 1 wt % CA-SDC dispersion confined within a PDMS mold (left). The sample after the freezing cycle (middle). The resulting physically crosslinked hydrogel formed after thawing (right). The F-T gelation process is driven by the ice-induced exclusion and mechanical entanglement of CA-SDCs at ice crystal interfaces, forming a robust, continuous network. (**B**) Images of the initial 1 wt% CA-SDC dispersion in bulk solution (left). The physically crosslinked CA-SDC hydrogel after a single F-T cycle (middle). The porous cryogel obtained after sequential solvent exchange and vacuum drying (right), demonstrating the stability and structural integrity imparted by the F-T process.

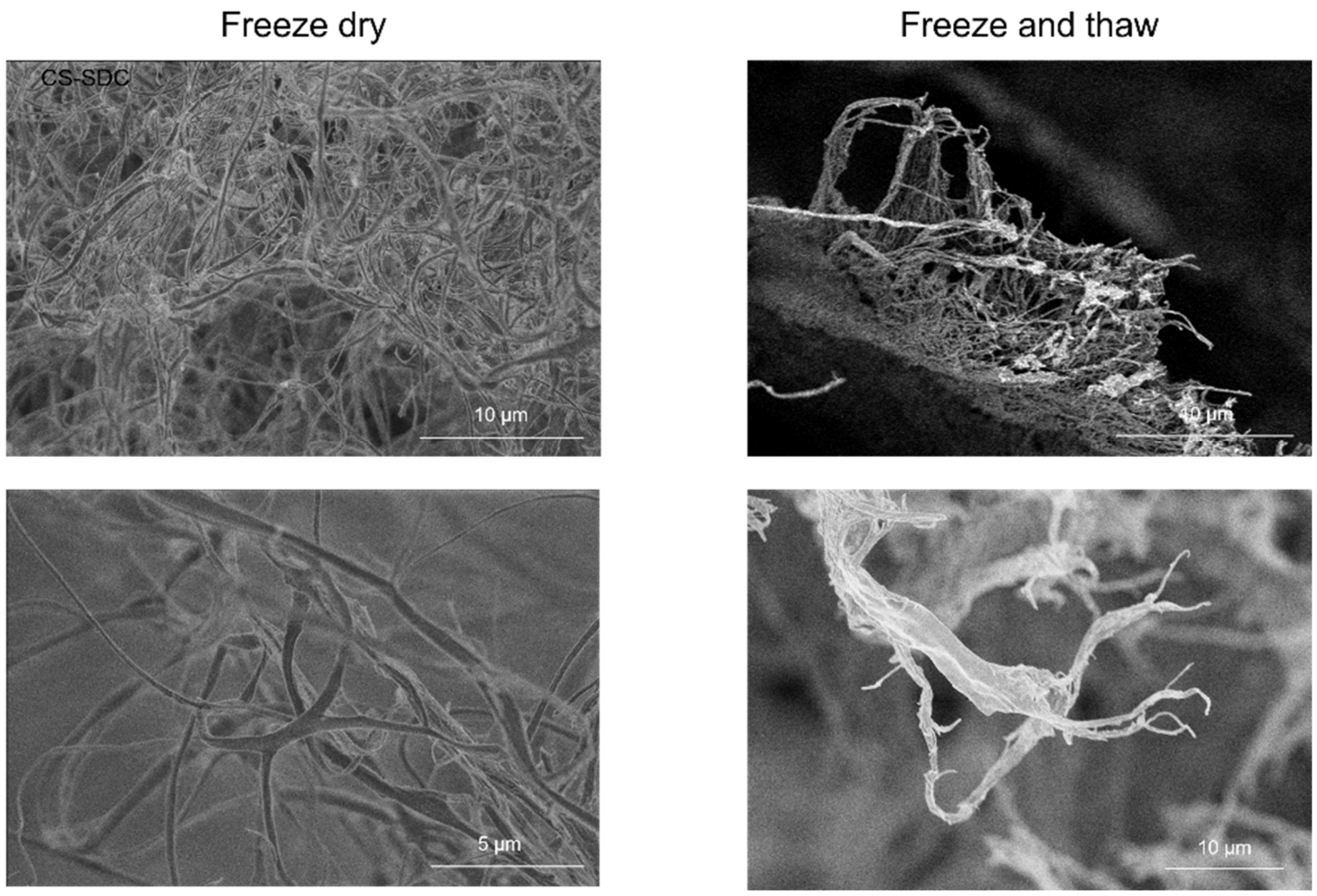

Fig. S3. SEM images of CA-SDCs after freeze-drying or F-T treatment. Freeze-dried CA-SDCs (left) show limited aggregation due to rapid freezing in liquid nitrogen, limiting ice crystal growth. In contrast, F-T treatment at -30 ºC (right) allows slower ice crystal growth, promoting colloidal confinement and enhanced interparticle interactions that yield a mechanically robust network.

Structural differences in F-T induced pore morphology of CA-SDC and CS-SDC hydrogel

F-T treatment applied to both types of SDCs results in the formation of fibrillar networks with large, interconnected pores and defined pore walls. The general morphology of CA-SDC and CS-SDC hydrogels appears similar, as the pore structures replicate the shape of growing ice crystals, which displace SDC fibrils into pore walls during freezing. While the F-T method is applicable to both CA-SDC and CS-SDC systems, only CA-SDCs consistently form thin, rigid, and sheet-like pore walls. Under identical SDC concentration, CA-SDCs produce denser and more compact wall structure, whereas CS-SDCs produce thicker, more porous walls composed of loosely entangled SDC fibrils. We hypothesize that the CS-SDC fibrils are more compliant and better accommodate ice crystal growth during the freezing phase, leading to less compression and lower wall density.

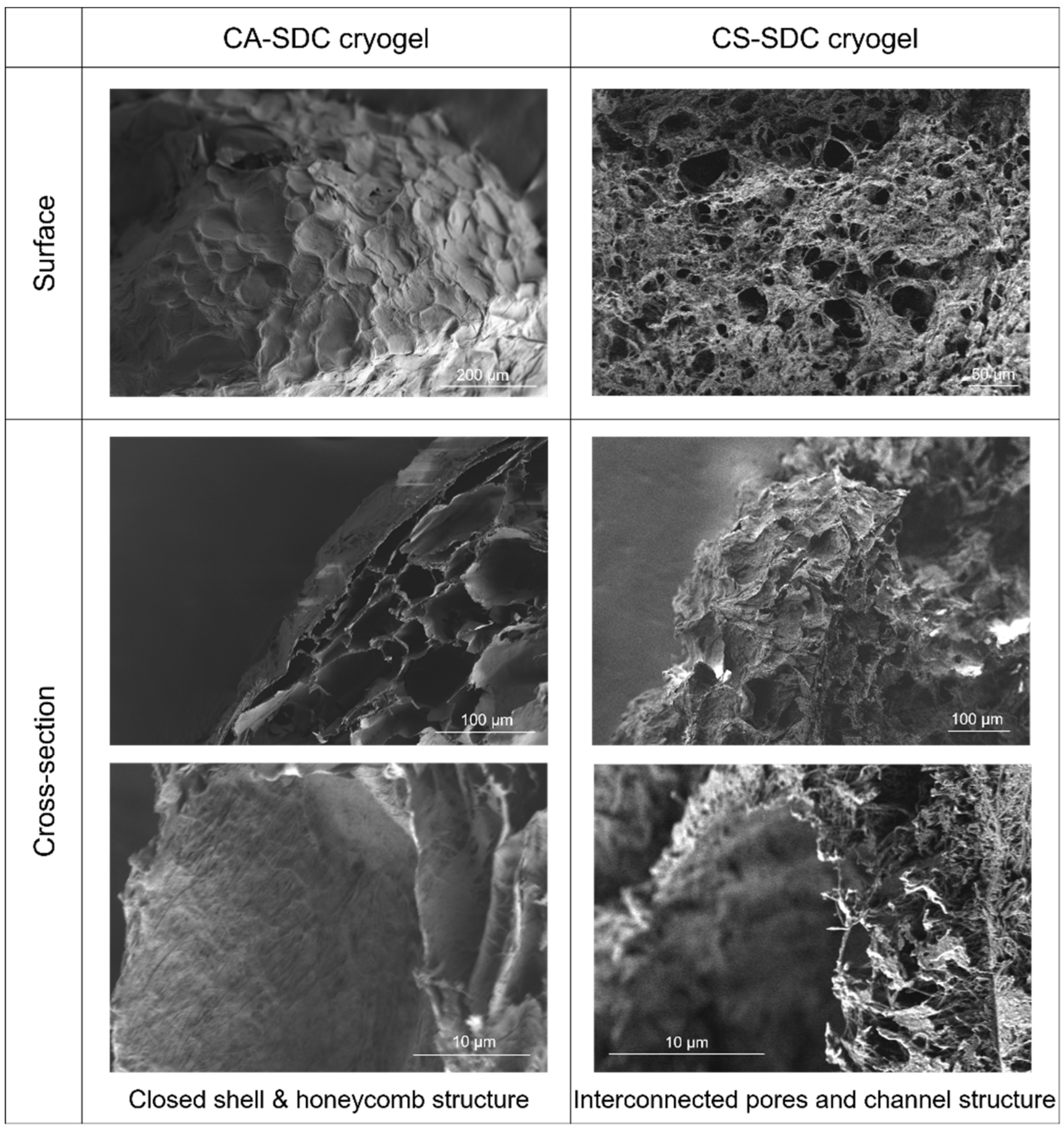

Fig. S4. SEM images of CA-SDC (left) and CS-SDC (right) cryogels. CA-SDCs form rigid, thin, sheet-like pore walls, whereas CS-SDCs form thicker, more porous walls composed of loosely entangled fibrils. Note the correspondence of some of the images to Fig. 3B.

Integrating mechanical stability and electrostatic adsorption for broad-spectrum NP and MP capture

As shown in Fig. S4, CA-SDCs formed hydrogel with rigid, thin, and densely packed pore walls, whereas CS-SDCs produced thicker, more porous walls composed of loosely entangled fibrils. This structural difference results in higher tensile strength prior to fracture for CA-SDC hydrogels compared to their CS-SDC counterparts (Fig. S5A).

To evaluate the influence of SDC material on MP adsorption, we assessed the polystyrene (PS) bead capture efficiency of both hydrogel types. Same concentrations of CA-SDC and CS-SDC hydrogels were introduced into the PS bead suspensions. The CS-SDC hydrogels, which have positive surface charge, demonstrated significantly higher adsorption efficiency than the negatively charged CA-SDC hydrogel. This observation supports the hypothesis that electrostatic interactions play a major role in the adsorption of microbeads onto the hydrogel surface.

These findings highlight the need for a dual-layer mesh composed of two distinct materials to achieve broad-spectrum NP and MP capture from aquatic environments. The mechanically robust CA-SDC hydrogel core provides structural support and enables passive filtration of larger MPs, while the fibrillated CS-SDC outer layer enhances adsorption of smaller NPs and MPs through van der Waals and electrostatic interactions.

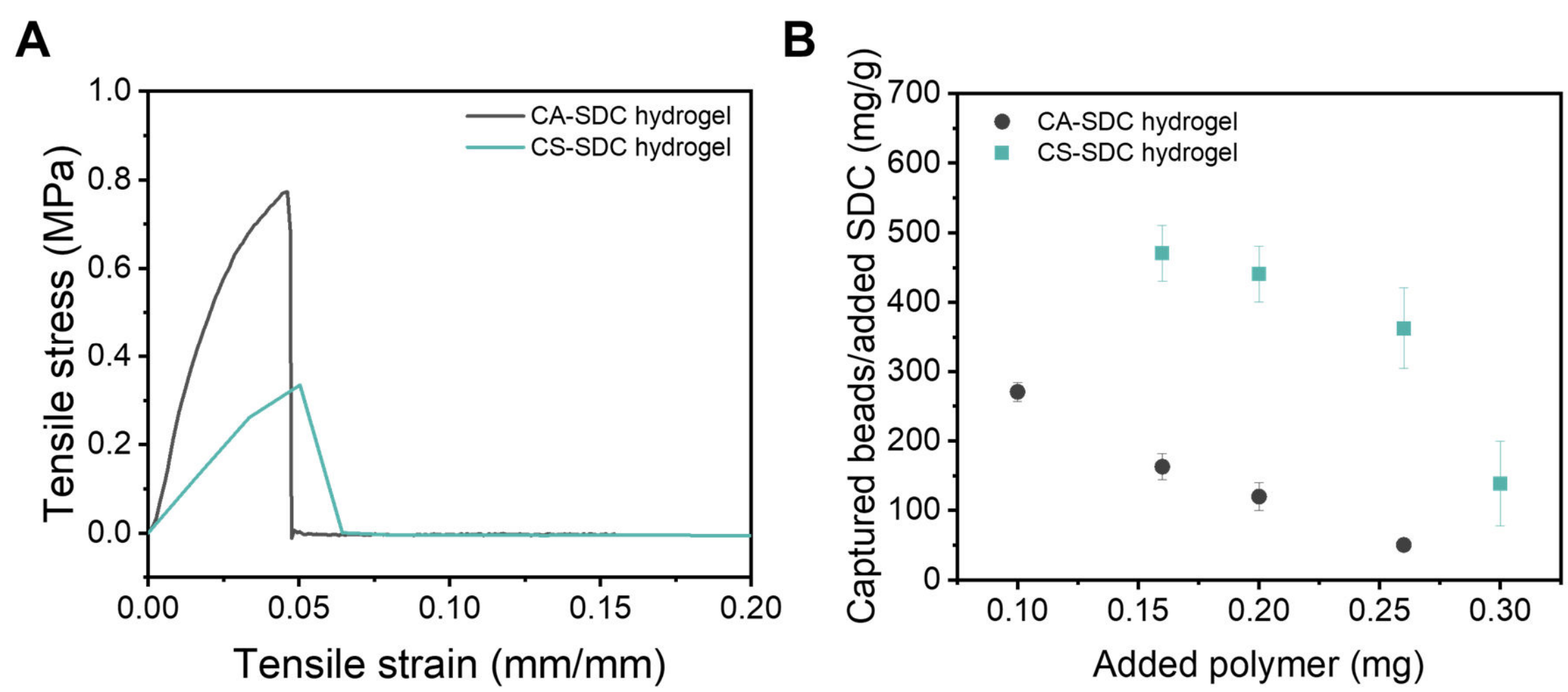

Fig. S5. Comparison of mechanical properties and MP capture performance between CA-SDC and CS-SDC hydrogels. (**A**) Tensile stress-strain curve of CA-SDC and CS-SDC hydrogels. (**B**) Efficiency of PS bead capture by spherical hydrogels composed of either CA-SDCs or CS-SDCs.

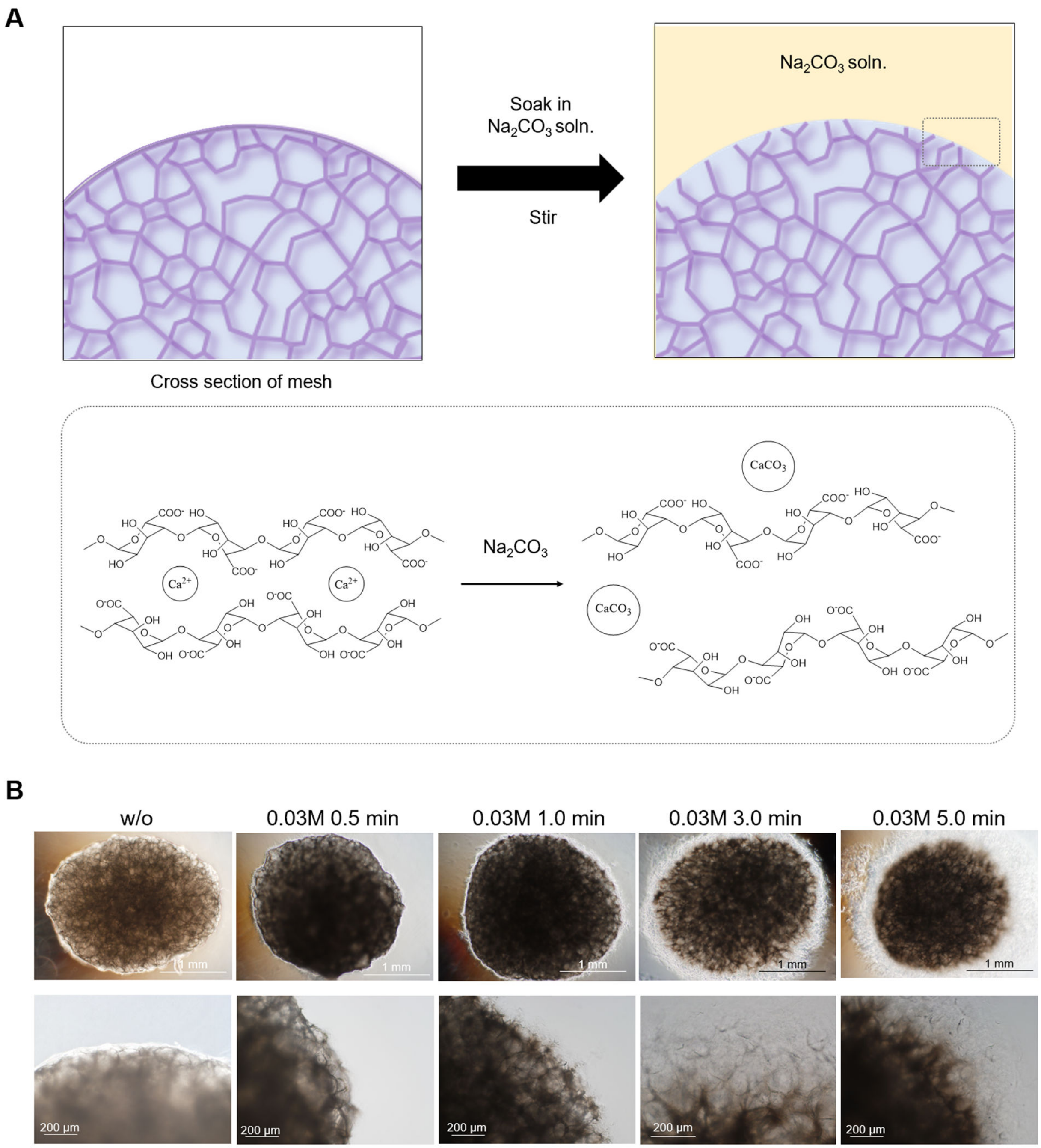

Fig. S6. Surface modification of CA-SDC hydrogels to create an outerlayer with "fluffy" morphology. (**A**) Schematic illustration of the surface modification process. The rigid surface of the CA-SDC hydrogel is partially degraded in sodium carbonate (Na_2CO_3) solution, where carbonate ions disrupt the ionic crosslinking of alginate by forming insoluble calcium carbonate. (**B**) Cross-sectional optical microscopy images showing the time-dependent surface changes of CA-SDC hydrogels immersed in 0.03M Na_2CO_3 solution. Note the correspondence of some of the images to Fig. 4A.

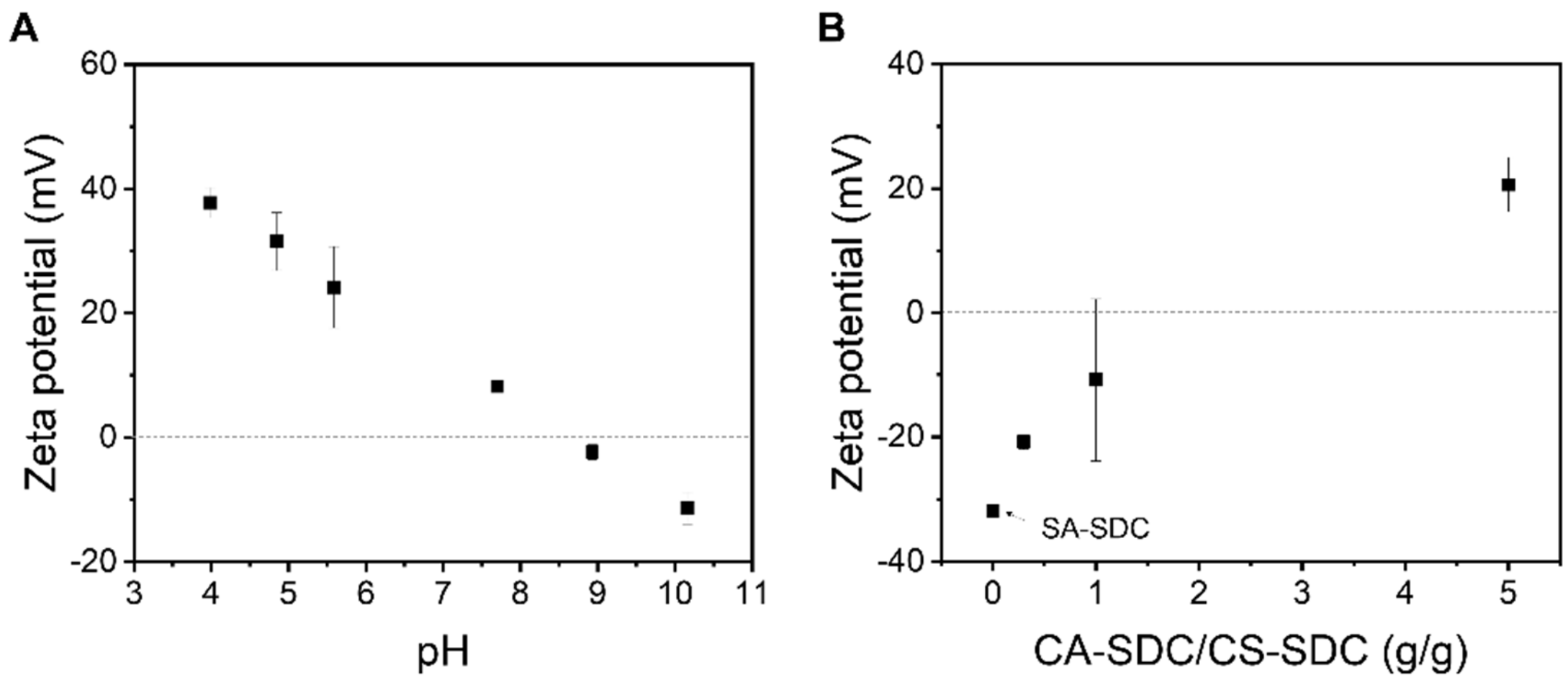

Fig. S7. Zeta potential characterization of hydrogel surfaces. (**A**) Surface zeta potential of CA-SDC hydrogels as a function of pH. (**B**) Surface zeta potential of CS-SDC/CA-SDC combined clusters.

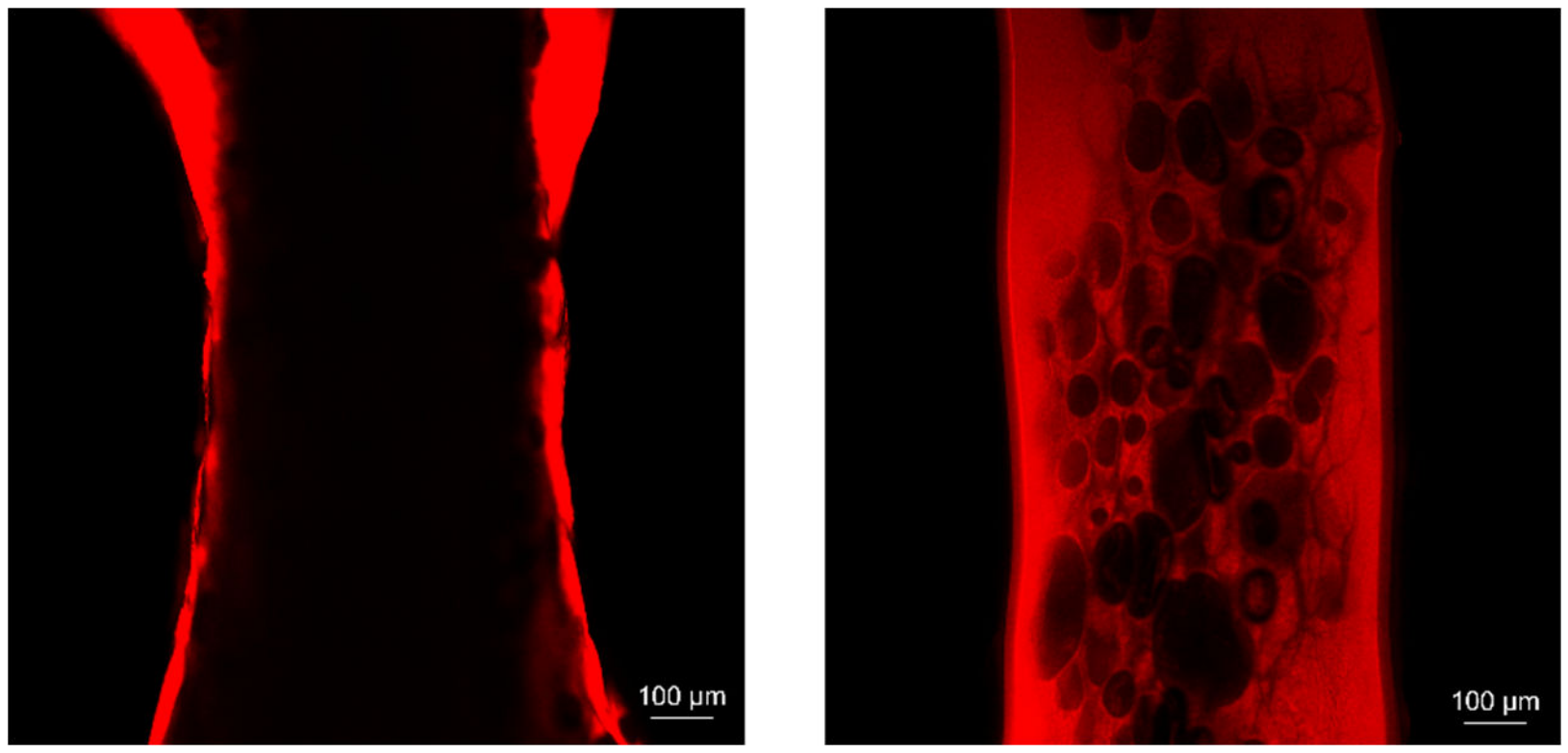

Fig. S8. Origin of mesh buoyancy. Confocal fluorescence microscopy of the macroscopic mesh (measured density of 0.0535 g/cm³) immersed in Rhodamine B-dyed water for 1 hour (left) and 24 hours (right). The cross-sectional images reveal numerous unstained void spaces within the structure, suggesting that air bubbles are stably trapped within the pore walls of the hydrogel network. Such trapped air is the likely origin of the buoyancy responsible for the mesh's flotation.

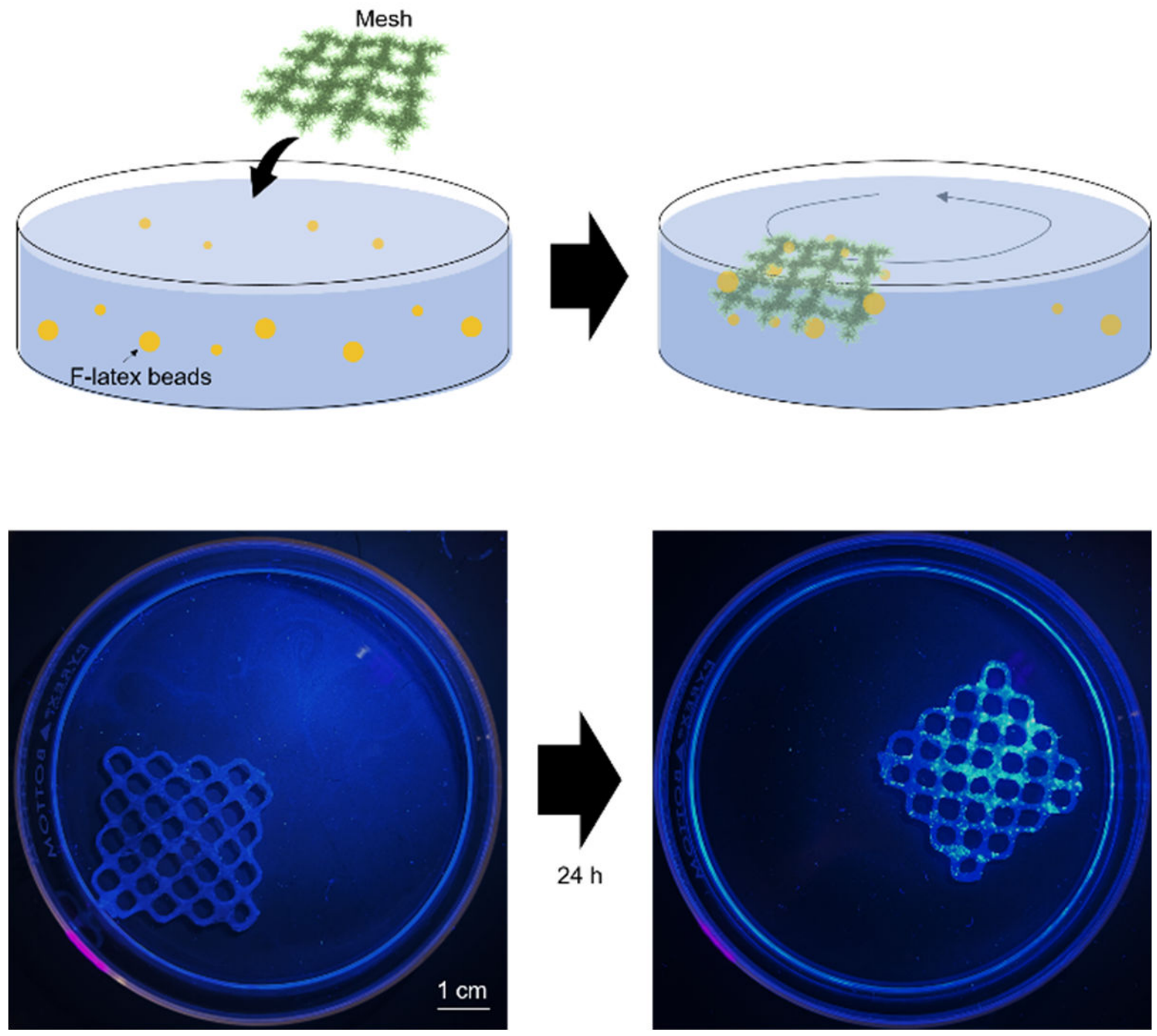

Fig. S9. Schematic and demonstration of MP capture test. Conceptual illustration of the measurement process (top). Experimental images showing capture of fluorescent dyed latex beads (bottom).

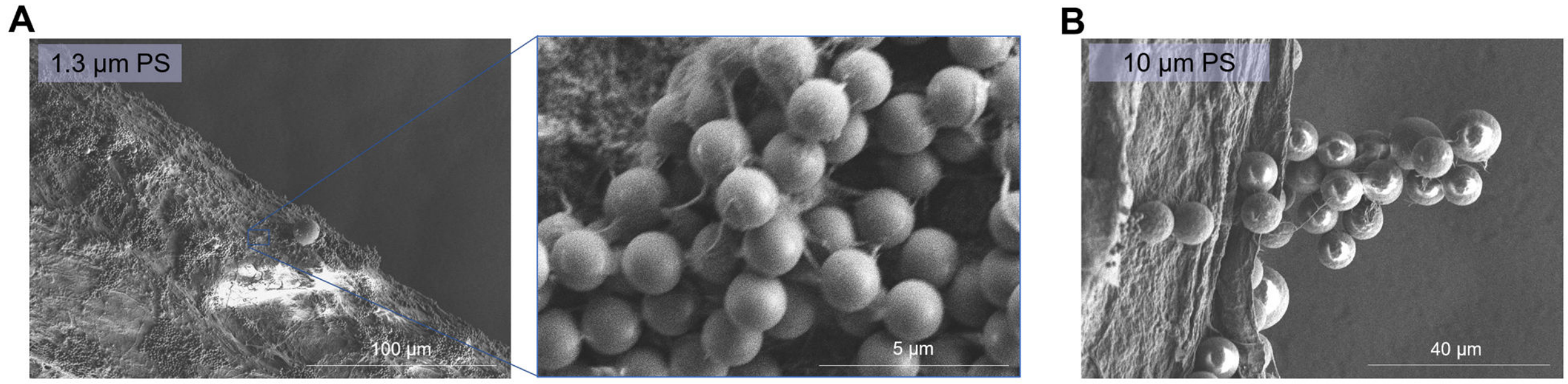

Fig. S10. SEM images of PS bead capture on the mesh. (**A**) 1.3 µm PS beads adsorbed onto the fibrillary surface. (**B**) Numerous 10 µm PS beads stacked into a multilayer cluster on SDC fibrils, demonstrating the tendency of smaller particles to form aggregates that enhance capturing efficiency.

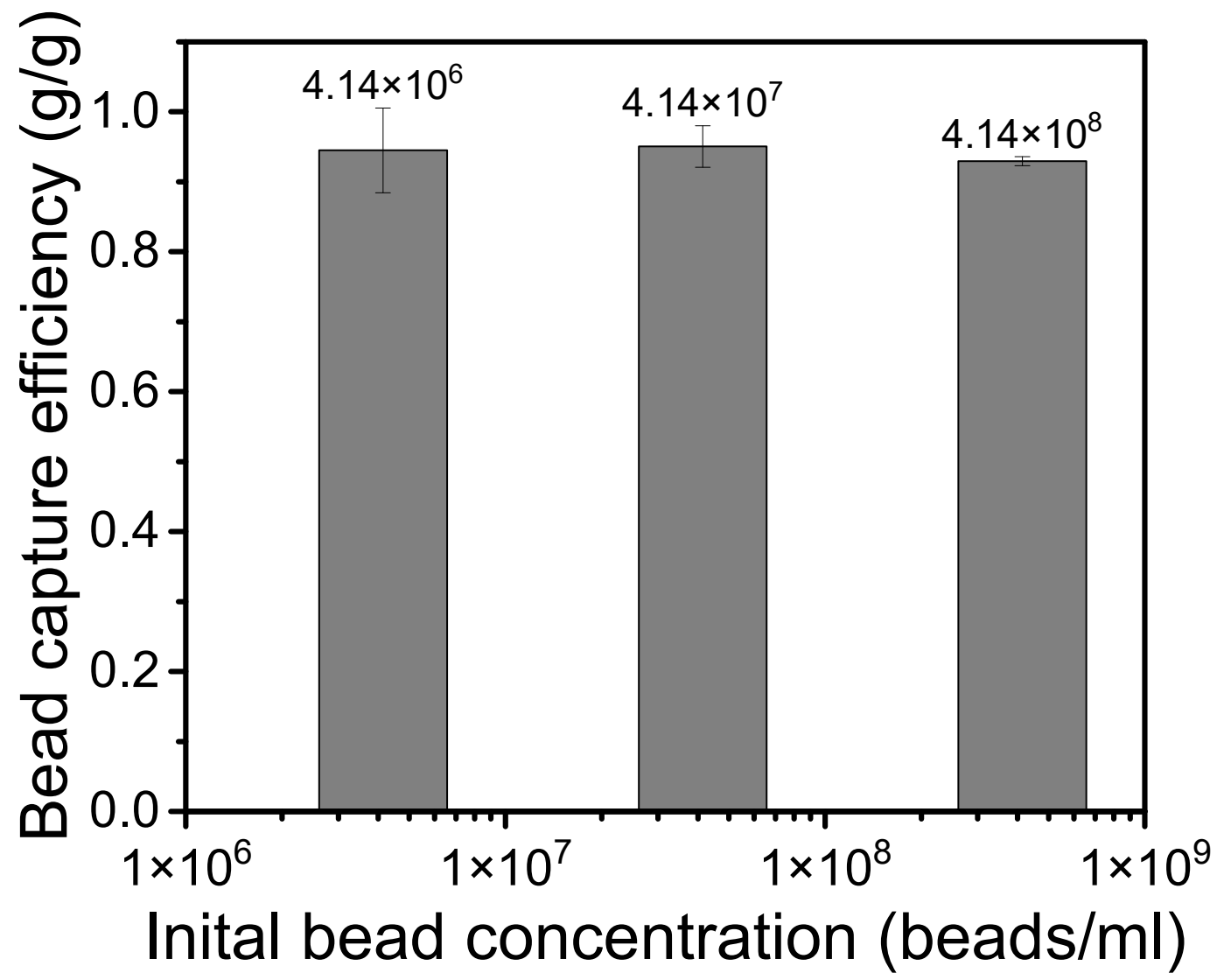

Fig. S11. Effect of initial MP concentration on capture efficiency. The capture efficiency of the biomimetic mesh (5 mg solid content) was evaluated across a range of initial concentrations of 1.3 µm PS beads. The mesh consistently achieved over 95% removal efficiency across all tested concentrations.

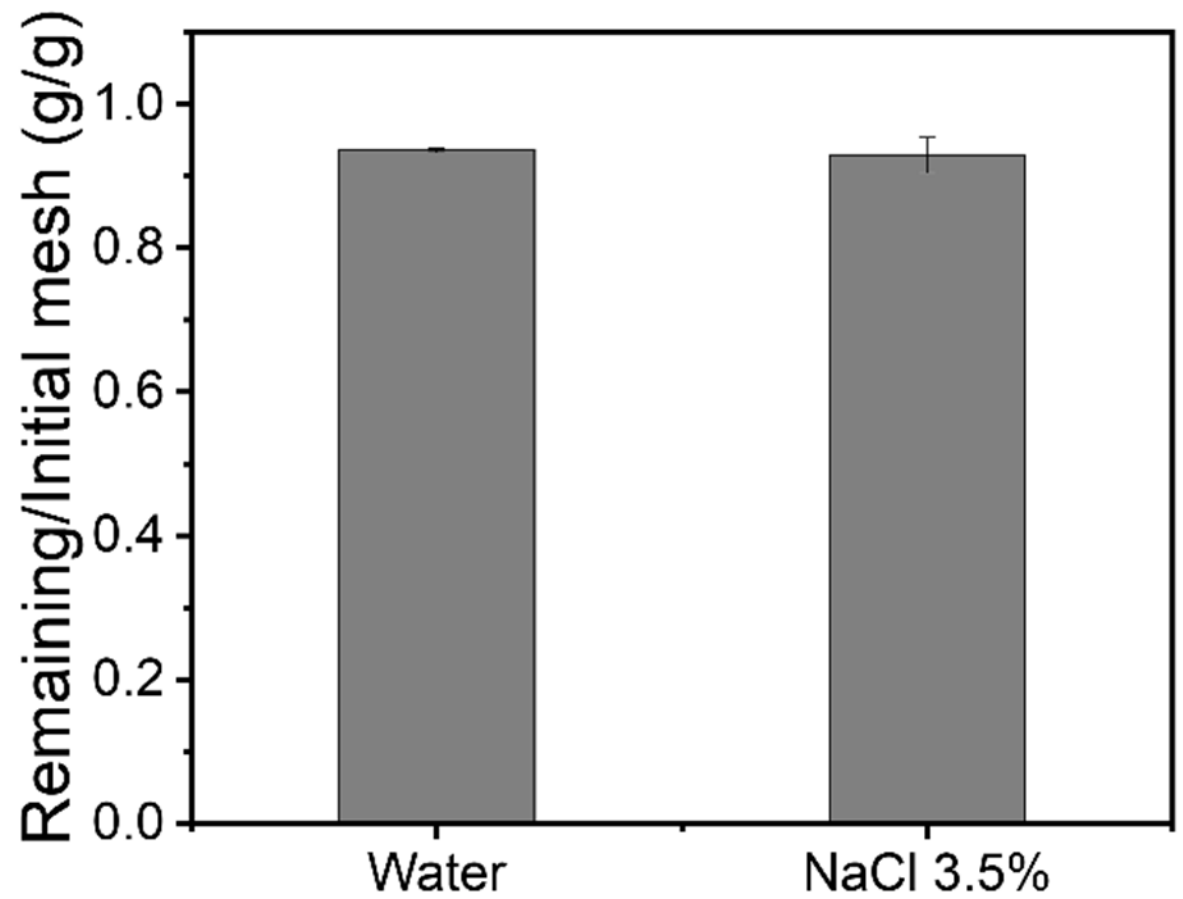

Fig. S12. Mechanical robustness of the mesh in pure water and saline medium. Comparison of the mass loss of the mesh under continuous agitation in DI water and saline water (3.5 wt% NaCl) using orbital shaker at 100 rpm for 24 h. This test evaluates environmental stability under intensive liquid shear conditions.

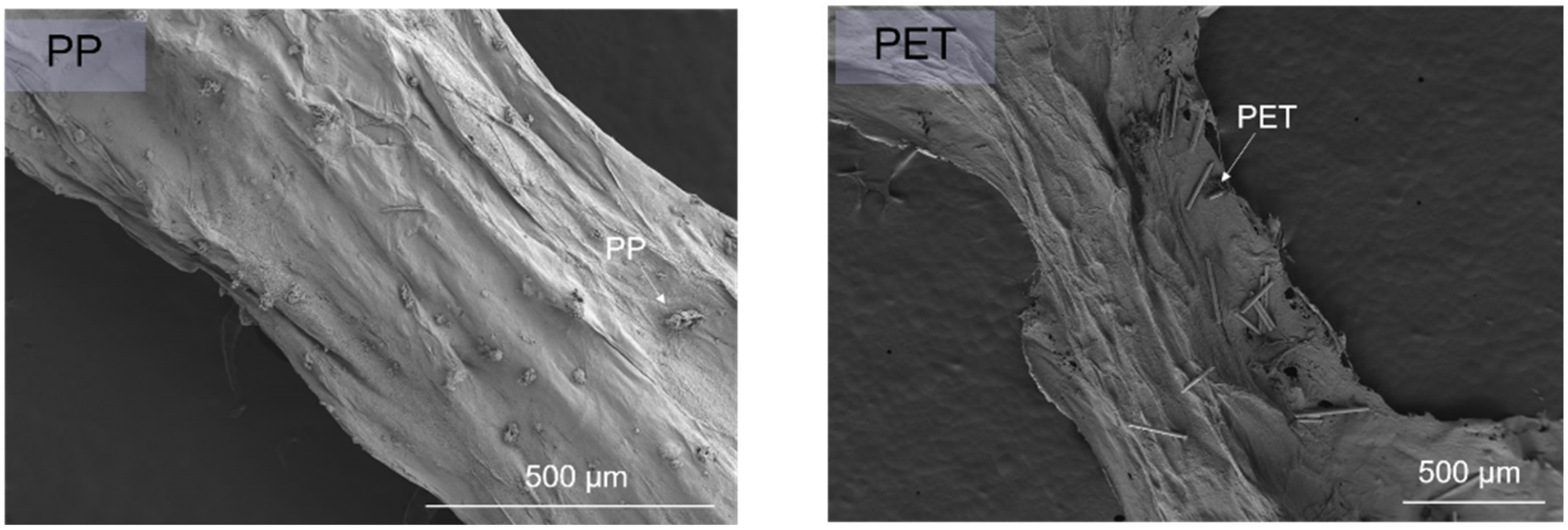

Fig. S13. SEM images of MPs with different polymer compositions captured on the mesh. PP (left) and PET (right) particles are trapped onto the fibrillary mesh surface.

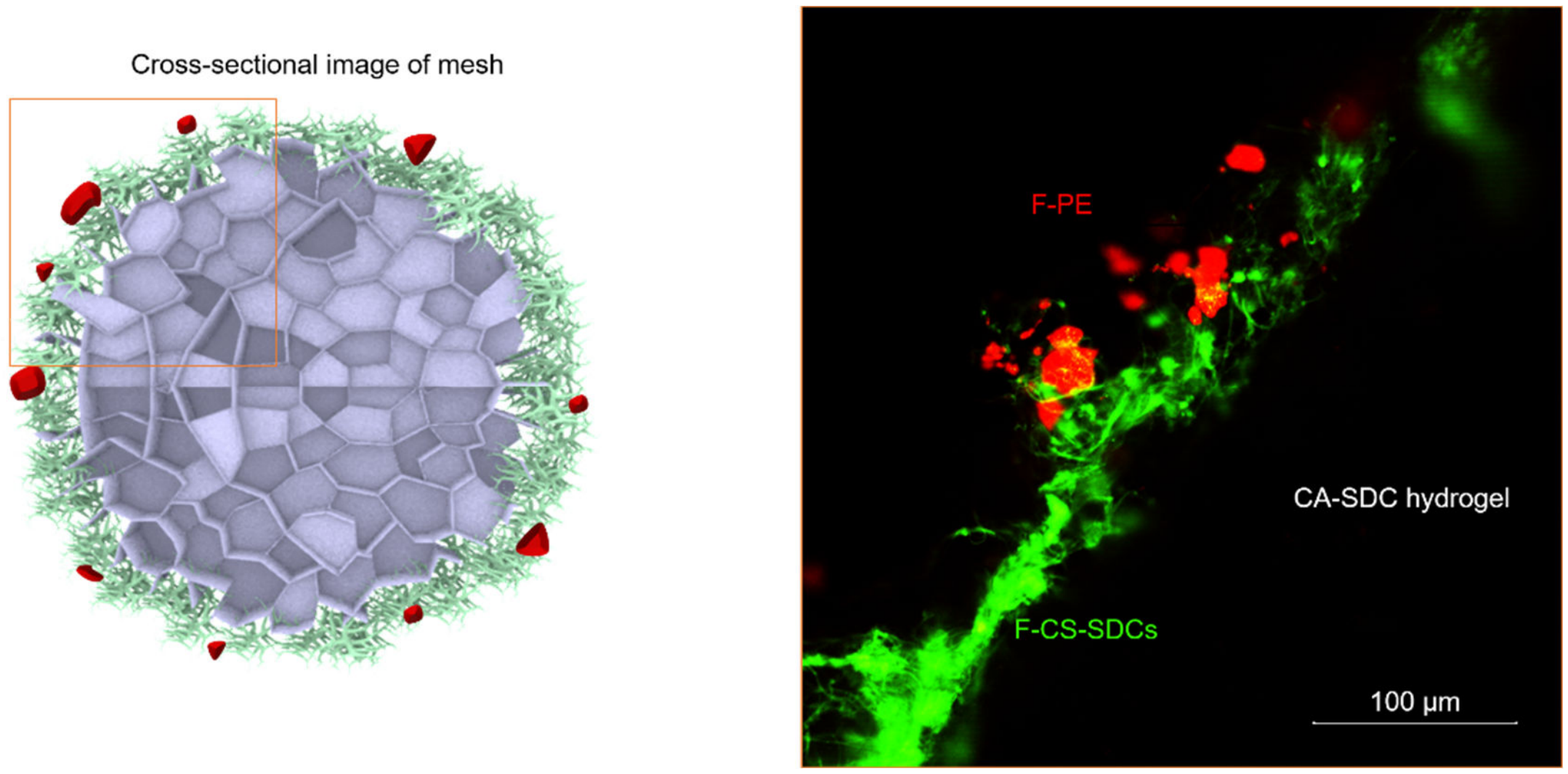

Fig. S14. Confocal microscopy of MP adsorption within the bilayer mesh. Cross-sectional images of CA-SDC hydrogels coated with CS-SDCs (green, labeled with fluorescein isothiocyanate) capturing PE MPs (red, labeled with Nile red), demonstrating that the particles are entangled and adsorbed on the fibrillar outerlayer.

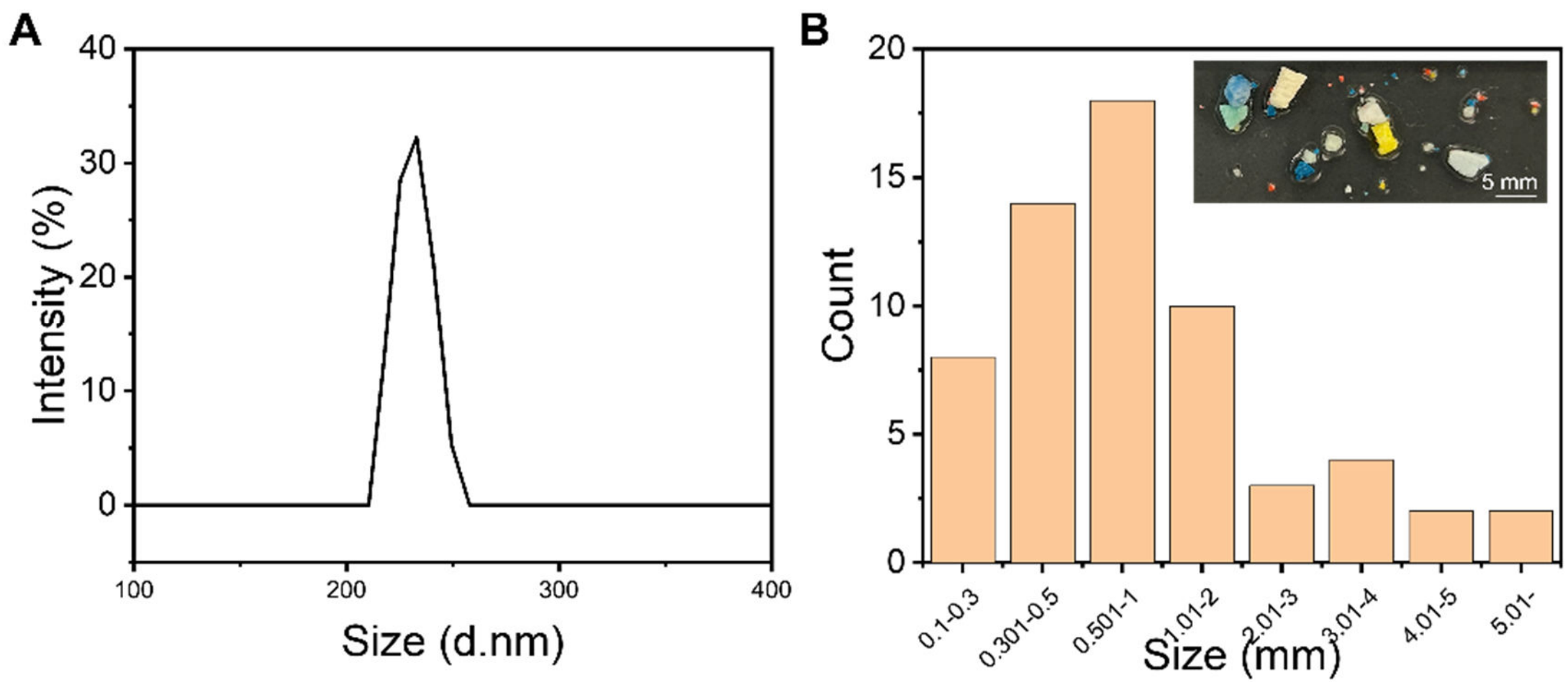

Fig. S15. Size distribution characterization of the environmental MP samples. The highly heterogeneous environmental samples were characterized by dividing them into two fractions: (**A**) The size distribution of the floating MP fragments, analyzed via optical imaging (ImageJ), showing a broad size range spanning from ~100 µm up to >5 mm. (**B**) Dynamic light scattering (DLS) analysis of the dispersed particle fraction. Measurements were conducted using PS parameters due to its environmental prevalence and near-water density, revealing a dominant submicron population distributed primarily between 200 and 300 nm.

Surface modified latex	Diameter (μm)	Density (g/cm3)	Initial bead concentration (#/ml)	
w/o surface modification	0.989		8E+07	Polysciences, Inc.
Sulfate latex	1.3		8E+07	Invitrogen
Carboxylate latex	1	1.05	8E+07	Sigma Aldrich
Amine latex	1		8E+07	Sigma Aldrich
Amidine latex	0.97		8E+07	Invitrogen

Table S1. Physical properties of PS latex beads with different surface modification used for MP capture efficiency measurements.

Diameter (µm)	Surface type	Initial bead concentration (#/ml)	Initial bead concentration (mg/ml)	
0.3	latex	6.7367E+09		Duke Scientific Corporation
1.3	sulfate latex	8.2791E+07		Invitrogen
5	sulfate latex	1.4551E+06	0.1	Invitrogen
9.4	sulfate latex	2.1899E+05		Invitrogen
100	latex	1.8189E+02		Alpha Nanotech Inc.

Table S2. Physical properties of PS latex beads with different sizes used for MP capture efficiency measurements.

Type of plastic	Shape	Size range (μm)	Zeta-potential (mV)
Polystyrene (PS)	Microsphere	9.4	-21.48
Polyethylene (PE)	Irregular shape	2.1-12.6	-29.73
polyethylene terephthalate (PET)	Rod	Width 11-15<br>Length 60-420	-9.74
Polypropylene (PP)	Irregular shape	10-107	-15.1

Table S3. Physical and surface properties of MPs from different polymers used for MP capture efficiency measurements. The size distribution of NMPs was determined by SEM (using ImageJ).

Captions of Movies S1 and S2

Other Supplementary Materials for this manuscript include the following movies:

Movie S1. Demonstration of the capture of a set of various macroscale particles by a fibrillar SDC mesh, followed by manual removal from water. Playback speed is 5× faster than real time

Movie S2. Demonstration of adsorption of various MPs by the SDC-based mesh under rotational mixing. Playback speed is 10× faster than real time.
